# Congenital Scoliosis: A Comprehensive Review of Diagnosis, Management, and Surgical Decision-Making in Pediatric Spinal Deformity—An Expanded Narrative Review

**DOI:** 10.3390/jcm14228085

**Published:** 2025-11-14

**Authors:** Paweł Grabala

**Affiliations:** Department of Neurosurgery, Polish-Mother’s Memorial Hospital Research Institute, Rzgowska 281/289, 93-338 Lodz, Poland; pgrabala@wp.pl

**Keywords:** posterior spinal fusion, congenital scoliosis, vertebral malformations, pediatric spinal deformity, hemivertebra resection, growing rods, posterior vertebral column resection (PVCR), thoracic insufficiency syndrome

## Abstract

Congenital scoliosis is a complex spinal deformity caused by abnormal vertebral development during embryogenesis, occurring in roughly 0.5–1.0 per 1000 live births. It encompasses a wide spectrum of anomalies that arise from failures in vertebral formation or segmentation, or mixed defects during the fourth to sixth weeks of gestation. Managing this condition requires careful consideration of growth dynamics, associated systemic anomalies, and individualized decisions regarding surgical timing. In this review, current evidence on the epidemiology, pathophysiology, diagnostic strategies, and treatment of congenital scoliosis is synthesized, with special attention to surgical decision-making between hemivertebrectomy and growth-preserving methods such as growing rods. Recent surgical techniques—including magnetically controlled growing rods and posterior vertebral column resection—have expanded therapeutic options, while greater recognition of the psychosocial impacts has underscored the importance of family-centered care. Successful management relies on striking a balance between early deformity correction and preserving long-term spinal and thoracic growth. Multidisciplinary collaboration and thorough family counseling remain central to optimizing both structural and psychosocial outcomes.

## 1. Introduction

Among pediatric spinal deformities, one of the most demanding conditions to correct is congenital scoliosis, necessitating decisions that judiciously weigh prompt correction against the potential costs of future growth [1]. Whereas idiopathic scoliosis occurs in otherwise typical spines, developing as they will, congenital scoliosis is caused by structural deformities present at birth [2]. Such vertebral anomalies pose distinct biomechanics and growth challenges that essentially define the course of treatment [2]. Congenital scoliosis (CS) is a spinal deformity caused by spinal malformations [3]. The progression as well as the corrective treatments differ based on the patient’s as well as the curve’s traits [3]. The condition is rarely isolated. Many children with congenital scoliosis also present with syndromic associations and multisystem anomalies, making comprehensive evaluation and coordinated interdisciplinary care essential [3,4,5,6]. In recent decades, the management philosophy has shifted from primarily corrective surgery toward growth-preserving techniques. This shift reflects recognition of the critical role of thoracic growth in pulmonary development and an improved understanding of the natural history of congenital anomalies [5,6,7].

Contemporary management integrates advanced imaging, genetic counseling, and multidisciplinary expertise to develop individualized treatment plans. These plans must account not only for the spinal deformity itself but also for associated systemic conditions and the psychosocial impact on patients and families [6]. Decision-making is therefore multifactorial, involving considerations of anomaly type, progression risk, patient age, growth potential, and family preferences, all weighed against the benefits and risks of available surgical options [8,9,10].

## 2. Methods

The aim of the present narrative review was a comprehensive synthesis of existing understanding of CS in the pediatric population, with specific reference to surgical treatment modalities. As well as operative management, the review also covers the epidemiology, etiology, and genetic pathogenesis of CS and its related congenital anomalies. A non-systematic, thematic literature review was carried out utilizing the Consensus academic search engine (https://consensus.app accessed on 30 August 2025) and PubMed/MEDLINE databases. The searches were narrow-ended specifically on studies with a focus in human subjects in the pediatric population. To capture the multifaceted nature of congenital scoliosis, the literature was searched in five core domains: epidemiology and incidence, etiology and gene pathogenesis, comorbid disease and syndrome, and techniques and outcomes in the surgical treatment of the condition. Results were supplemented with citation chaining and reference truncation in high-quality review articles. The selection of studies was made after title and abstract screening with subsequent full-text analysis. Extracted information was organized thematically according to the following domains: epidemiological information such as prevalence, age at presentation, and gender distribution; etiological determinants such as the type of vertebral malformations, embryological origin, and described mutations, including TBX6, DLL3, and MESP2; comorbid disease, with specific reference to cardiac, genitourinary, and neural axis anomalies; techniques and outcomes in surgery, such as indications, procedure descriptions, and age at surgery; outcomes such as Cobb angle correction and growth and quality of life; and complications such as neurological damage, implant failure, infection, and revision surgery. Due to the nature of the review being a narrative style, no formal quality appraisal was employed. This review was specifically undertaken in a narrative style in order to facilitate flexible inclusion of a large range of topics. In contrast to a systematic review, it does not attempt exhaustively numerating the evidence but instead summarizes, contextualizes, and critically synthesizes existing understanding—with a specific focus on surgical treatment modalities in congenital scoliosis.

## 3. Epidemiology and Associated Anomalies

### 3.1. Incidence and Population Burden

Congenital scoliosis affects approximately 0.5 to 1 in every 1000 live births [6,11,12,13], accounting for nearly 10% of all pediatric scoliosis cases [14]. Among affected individuals, about a quarter will exhibit no curve progression, another 25% will experience slow deterioration over time, and roughly half will eventually require surgical treatment [15]. A population-based study using data from the Korean Statistical Information Service reported a prevalence of 3.08 cases per 100,000 individuals over a five-year period, highlighting the rarity of this condition in the general population [3,7,16]. In addition to scoliosis, congenital spinal deformities can also manifest as kyphosis—an abnormal backward curvature of the thoracic spine—or lordosis, which refers to an exaggerated forward curve typically in the lumbar or cervical regions. These forms occur less frequently than congenital scoliosis [17,18].

Despite these classifications, many cases go unnoticed in early life. Asymptomatic individuals may not receive a diagnosis until incidental findings of congenital vertebral malformations (CVMs) appear on radiographic imaging conducted for unrelated concerns [13,14,15,18]. Some recent studies based on population sampling with the use of accidental screening in the context of the use of emergency abdominal–chest radiographs found the incidence of congenital scoliosis to be 89 out of 50,426 infants, reflecting a prevalence of 1.8 per 1000. This implies that some instances might go unnoticed during early infancy [2]. Such studies demonstrate the value of systematic screening with the use of a lateral view in the right situation as well as the role of early identification measures.

At an embryological level, somites derive from the paraxial mesenchyma within the trilaminar germ disk. Somites harbor the precursor somitic cells that give rise to the backbone as well as to the striated musculature of the trunk. Somitogenesis happens between the 20th and 35th day following conception, with disruption of the mechanism playing a role in the causation of CVM causative of CS [12]. The procedure implicates signaling pathways including Notch1, FGF, and HOX, as well as Wnt [7,19,20].

Owing to the involvement of CS in a host of congenital syndromes, such as CHARGE, Klippel–Feil and VACTERL [7,12,16,20,21], there are thought to be multiple genetic factors that contribute to CS. Instances of one among a pair of monozygotic twins exhibiting CVM with the other being asymptomatic have been observed and have confirmed the role of environmental factors as well as genetic ones [15,16,17,22,23]. The prevalence is also geographic as well as ethnic in nature, with higher prevalence among some populations [17,24,25,26]. Associated anomalies are frequent, occurring in 60–70% of the patient incidence. The most frequent involve the genitourinary system (20–30%), the cardiovascular system (10–15%), and the central nervous system (15–20%) [27,28,29]. A well-recognized example is the VACTERL association (vertebral defects, anal atresia, cardiac anomalies, tracheoesophageal fistula, renal anomalies, and limb defects), which is frequently seen alongside congenital vertebral malformations [2,3,30].

### 3.2. Genetic and Developmental Factors

Recent molecular studies have identified several genetic loci linked to congenital scoliosis, including TBX6, MEOX1, NKX3-2, and various components of the Notch signaling pathway [11,12,14,15,19,20]. These genetic discoveries have significantly deepened our understanding of the developmental mechanisms behind vertebral malformations. Moreover, they hold promise for future applications in precision medicine and potentially informing individualized therapeutic strategies and improving genetic counseling protocols for affected families [7,23,31,32,33]. Importantly, recognizing hereditary risk factors allows for more accurate assessment of familial recurrence, especially in cases involving multiple affected siblings or multigenerational inheritance patterns.

The advancement of genomic technologies, particularly whole-exome sequencing (WES), has greatly enhanced our ability to uncover the genetic architecture of complex developmental anomalies. Mutations that disrupt the intricate genetic programming of embryogenesis can lead to various forms of congenital spinal deformity. Critical signaling pathways, such as fibroblast growth factor (FGF), Wnt, Notch, and others, play integral roles in different stages of vertebral formation [17,23,34,35]. These pathways are finely regulated through time-sensitive gene expression patterns that guide proper tissue differentiation and organ development.

At a higher level, these genes are controlled by regulatory elements that modulate their activation and suppression. Mutations in either the genes themselves or their regulatory mechanisms can interrupt normal developmental processes, leading to spinal malformations. Understanding this multi-tiered genetic regulation is essential for interpreting the origins of congenital scoliosis and related conditions [16,33,36].

Traditional classification systems, which primarily focus on anatomical outcomes of failed vertebral development, such as segmentation or formation errors, may no longer fully capture the biological complexity of congenital spinal deformities. A more comprehensive classification system is emerging, informed by genetic findings from technologies like WES [33,36,37,38,39].

This new framework could involve a dual classification system: developmental-stage-specific classification, which categorizes malformations according to the phase of vertebral development (e.g., formation vs. segmentation) in which disruption occurs, and pathway-specific classification, which groups deformities based on the disruption of specific molecular signaling pathways, such as Notch, FGF, or Wnt. Together, these two perspectives provide a more nuanced and biologically grounded approach to diagnosing, managing, and researching congenital spinal deformities. They not only aid in understanding disease mechanisms but also pave the way for targeted interventions and personalized care strategies in the future.

Environmental factors during the early course of pregnancy are also implicated. Maternal diabetes, use of certain medications, and deficiencies in nutrition have all been identified as musculoskeletal malformation associates [12,14,15,16]. Awareness of these factors is important not only during patient assessment but also as possible preventive measures in high-risk pregnancies. Although a direct link between gestational diabetes and congenital scoliosis has not been clearly established, studies have shown an association between gestational diabetes and musculoskeletal anomalies [14]. Acknowledging such risk factors is significant not just during patient assessment but also for possible prevention measures in high-risk pregnancies. The genetic etiology is poorly understood, yet exome sequences as potential associates of genes is proposed. For example, a compound inheritance of a null mutation along with a hypomorphic allele of the T-box 6 (TBX6) gene is implicated in 10% of sporadic CS cases [11,15,20]. Other studies have widened the mutational spectrum along with enhanced molecular diagnostic value. Furthermore, LFNG mutations have also been shown to correlate with CVM, possibly leading to a spectrum of presentation including CS along with SCD (spondylocostal dysostosis) [11,16,20]. Autosomal dominant trait patterns related to CS have also been recognized in the FBN1 gene, which is similarly known to be the cause of Marfan syndrome along with a continuum of additional syndromes of skeletal dysplasia. FBN1 could possibly be related to monogenic CS [40]. There are also some other genes reported in the literature thought to be connected to CVM that still deserve to be investigated. Other congenital conditions that are linked to CS include connective tissue disorders like Beal’s syndrome or Marfan syndrome, congenital muscular dystrophy, hypotonia, and spinal cord deformities, as well as discrepancies in leg length [13,16,17,33,37,38,39].

Environmental factors could be significant in the etiology of CS. Numerous exposures have been shown to lead to CS such as gestational diabetes [12,35] and hypoxia [12] as well as carbon monoxide exposure from cigarette smoke during the formation of somites, causing hypoxia as well as reactive oxygen species [12,35]. Additionally, long-standing febrile states as well as hyperthermia exposing the fetus to hot temperatures [12,15,17] are also causative of CVM. The same can be said about the use of antiepileptics [35,37,38,39], such as valproic acid [12], as well as with the consumption of alcohol, which is linked with the occurrence of Klippel–Feil syndrome [13]. Environmental toxins such as boric acid [7,20,21], as well as teratogenic factors, spinal tumors, and cancers [13,20,41,42] were found to be linked to CVM. Unlike in the case of idiopathic scoliosis, endocrine factors were not found to induce CS [22,43]. CS is also linked with rickets as well as with nutritional deficiencies, as well as with vitamin deficiencies [2,3,30]. There are various non-vertebral anomalies that are seen with CS, including genitourinary [32,41,42,44], musculoskeletal [3], cardiac [44], and rib anomalies [41]. Therefore, it is necessary to conduct renal as well as cardiac ultrasound/MRI [42,44], musculoskeletal assessment [45], and vital capacity screening (since the functioning of the lungs may be impaired by interconnections between the ribs, as well as vertebral anomalies) [42] in such individuals. The incidence of these defects will in no way impact the progression of vertebral abnormalities, but they could impact surgical procedures.

## 4. Pathophysiology and Classification

### 4.1. Embryological Development and Malformation Patterns

Vertebral morphogenesis typically unfolds during the fourth through sixth gestational weeks via sequential processes of segmentation, chondrification, and ossification [1,2,3]. Three principal developmental phases characterize vertebral formation: mesenchymal, cartilaginous, and osseous phases. Spinal development encompasses notochordal formation, somitic differentiation, sclerotomal migration, chondrification, and vertebral ossification [2]. Vertebral morphogenesis initiates with notochordal establishment, wherein the paraxial mesoderm transforms into the axial skeletal framework [46]. Somites—bilaterally paired segmented formations that generate vertebrae—arise from paraxial mesenchymal tissue. The ventral somitic compartment produces the sclerotome, which develops into vertebrae and costal elements, while the dorsal portion generates the dermomyotome, subsequently forming dermatome and myotome components that constitute integumentary and muscular tissues [47,48,49].

Sclerotomal formation occurs under regulation by Sonic hedgehog (SHH) protein secreted from the neural tube’s floor plate and notochordal structures [33,47,48,49]. During the fourth embryonic week, sclerotomal cells migrate circumferentially around notochordal and neural tube structures, ultimately establishing cartilaginous tissue formation. SHH expression within the vertebral component of developing spinal elements activates the PAX1 transcription factor (TF), essential for sclerotomal cells to establish ventral and dorsal cellular identities [33,36,49]. Collaborative function between the PAX1 protein and additional TFs, including PAX9 and MFH1 proteins, drives axial skeletal morphogenesis. PAX1 proteins orchestrate sclerotomal cell differentiation into chondrocytes responsible for cartilage formation and somitic segmentation [36]. Along the dorsal aspect of developing vertebrae, bone morphogenic proteins (BMPs) participate in osseous and cartilaginous tissue formation [50]. The combined effects of SHH and BMP signaling establish the dorsal and ventral vertebral components.

The subsequent vertebral developmental phase represents the cartilaginous stage, during which chondrification centers emerge in the sixth embryonic week. The terminal spinal developmental phase constitutes the skeletal stage, commencing at eight weeks and continuing until the mid-twenty-week stage. Between gestational weeks five through eight, sclerotomal cells undergo differentiation into chondroblasts. This cartilaginous vertebral model formation process operates under stringent HOX gene control. HOX gene regulation provides anterior–posterior positional specification during vertebrate development and ensures appropriate vertebral column regionalization [51,52]. HOX genes confer spatial identity to developing embryonic tissues throughout the organism. Mutations within these genes modify tissue identity within their expression territories, frequently affecting anterior regions.

In summary, HOX genes establish regional identity for developing vertebrae, whereas SHH signaling governs PAX1 protein expression within sclerotomal tissue. Congenital spinal deformities may arise from mutations affecting any of these genetic elements, potentially triggering cascading effects that disturb the equilibrium of these molecular interactions. Modified PAX1 expression levels resulting from disrupted SHH signaling may produce sclerotomal differentiation abnormalities. Likewise, alterations in HOX gene expression patterns may influence vertebral dimensions, morphology, and alignment through modified interpretation of PAX1 and SHH signals throughout vertebral development. The intricate molecular cross-talk among these genes emphasizes the critical importance of precise regulatory control during embryonic development to prevent congenital malformations. Disruptions in these processes lead to three primary types of malformations: failures of formation (e.g., hemivertebrae), failures of segmentation (e.g., unsegmented bars), and mixed anomalies that combine both [51,52,53].

Hemivertebrae, the most common formation defect, occur when half of a vertebral body fails to form. They can be fully segmented (with normal disc spaces above and below), partially segmented (with a disc space on only one side), or unsegmented (no disc spaces). Fully segmented hemivertebrae carry the highest risk of progressive deformity due to their growth potential [10,54,55,56].

**Figure 1:**
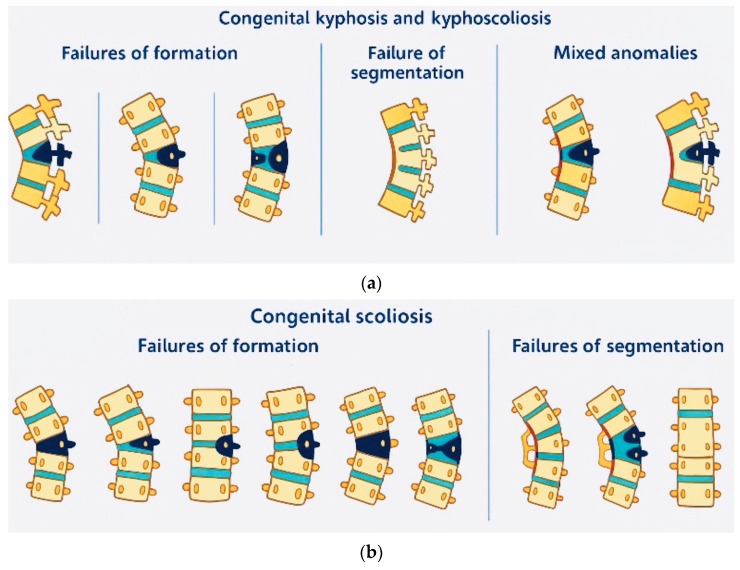
(**a**) Pathophysiology and classification of congenital deformities (kyphosis and kyphoscoliosis) adapted for the study [1,2,3,5,8]. (**b**) Pathophysiology and classification of congenital deformities (scoliosis) adapted for the study [1,2,3,5,8].

Segmentation failures occur when vertebrae fail to separate properly, leading to congenital blocks or bars [57]. A unilateral unsegmented bar creates tethering on one side of the spine, resulting in asymmetric growth and progressive curvature [58]. The combination of a hemivertebra and a contralateral bar is particularly severe, often progressing rapidly and necessitating early surgical intervention [1,2,3,7]. Pathophysiology and classification of congenital deformities are shown in Figure 1a,b.

Kawakami and colleagues introduced a revised classification framework for congenital spinal deformities, organizing these anomalies into four categories: formation failure, coupling failure, segmentation failure, and mixed failure [56]. Formation failure and segmentation failure maintain the same defining features originally described by Winter and colleagues [59]. This updated classification system distinguishes itself from earlier frameworks through the incorporation of a coupling failure category. Coupling failure represents aberrant somitic pairing, which leads to contralateral hemivertebrae development. Such malformations were previously categorized under formation failure. Mixed failure encompasses coupling failure combined with either segmentation or formation abnormalities. It should be emphasized that congenital spinal deformities demonstrate considerable heterogeneity, and classification schemes based on vertebral developmental failure patterns may lack completeness and accuracy. The classification systems developed by Winter et al. [59] and Kawakami et al. [24,56] have inherent limitations, particularly the inclusion of categories such as ‘mixed type’ and ‘mixed complex,’ which accommodate cases that cannot be categorized into other groups due to combined defects. The existence of these ‘mixed’ categories creates difficulties in treatment planning, prognostic assessment, and research endeavors. Consequently, there is a need to establish a classification methodology that adequately captures the multifaceted nature of congenital spinal deformities.

The examination should start with a review of the patient’s family history [13]. Obstetric history should be also assessed as well as current fetal imaging to investigate possible pre-natal vertebral defect [13]. The mother’s health issues and drug/substance use should also be mentioned [13]. The physical examination includes cognitive assessment [13], examination of height and weight, evaluation of the skin, neurological examination, evaluation of truncal as well as pelvic balance, and a search for spinal spine deformities as well as upper and lower limb asymmetry [60]. One of the crucial components of CS patient evaluation is thoracic insufficiency syndrome assessment with a thumb excursion test. Imaging examination begins with anteroposterior (AP) and lateral plain X-rays to document the Cobb angle of the curve to assess the CS and analyze its progression [35]. EOS imaging is recommended as an imaging technology that uses an ultra-low dose of radiation to create three-dimensional models from two planar images. Unlike a CT scan, EOS images are taken while the child is in an upright or standing position, enabling improved diagnosis due to weight-bearing positioning. CT scans along with 3D CTs are utilized to assess the anatomy to detect bony abnormalities and analyze the possibility of thoracic insufficiency syndrome [35] and ascertain the presence of intraspinal anomalies commonly seen with CS, with the highest incidence of abnormality being syrinx, diastematomyelia, and tethered cord [44]. They occur with a higher frequency in patients with catastrophic failure of segmentations, with a disproportionate number being seen among females [25] as well as among patient with anomalies of the ribs [41]. Owing to this high incidence rate, patients with CS should be evaluated with the use of MRI prior to surgical correction [44]. A tethered cord must be corrected prior to surgical scoliosis correction [3]. Signatures of these abnormalities are neurocutaneous markers and anomalies of reflex [3]. The vertebral abnormalities can occur in isolation or with other syndromes such as VACTERL syndrome [6]. To be able to categorize all the various vertebral abnormalities, they are first grouped into scoliosis due to longitudinal imbalance or scoliosis due to rotational imbalance [18]. The latter is then subdivided into spinal traction anomalies, spinal pushing anomalies, and mixed anomalies [18]. The longitudinal imbalance group is divided into four groups of scoliosis due to failure of segmentation, failure of formation, mixed defects, and complex unclassifiable defects [26,61,62]. Failures of formation comprise wedged vertebra, hemivertebra with varied levels of segmentation, and hemivertebral body [18]. Failures of segmentation comprise vertebral block or unilateral longitudinal bar [18,26,62], which might be as a growth tether [42]. Hemimetameric shift is the balance due to the occurrence of two contralateral hemivertebrae separated by one regular vertebra [3]. Such varied anomalies are mostly located at the apex of the curve, with the most frequent being the hemivertebra [18,25].

### 4.2. Growth Patterns and Progression Risk

The natural history of congenital scoliosis is variable, depending on anomaly type, location, and extent [63]. Unlike idiopathic scoliosis, which tends to accelerate during growth spurts, congenital curves may progress steadily throughout childhood [64]. Thoracic anomalies carry greater risks of progression and cardiopulmonary complications compared to lumbar anomalies [65].

Risk factors for rapid progression include multiple anomalies, mixed malformations, and lesions at the thoracolumbar junction [66]. Progression correlates with growth asymmetry between the convex and the concave sides of the curve and this growth happens mostly on the convex side [17,24,35]. It is known that 50% of curves advance quickly, 25% advance slowly and the other 25% do not advance at all. Progression happens at the ‘normal’ disc spaces, but the fused segments do not advance. The speed of advancement depends on the patient’s age, the apex’s location, the kind of the anomaly and the curves’ characteristics [3,26]. Age: the progression is the highest prior to 5 years of age and during the growth spurt during the patient’s teenage years (aged between 11 and 14 years old) [35]. Curves that are clinical prior to 10 years of age have a poor prognosis because of growth potential and if deformities are obvious during the patient’s first year of life then the worst prognosis has to be foreseen [3,6,42]. Location of the apex: the thoracic upper area curves show the lowest progression, whereas in the mid-thoracic area it is quicker, while the fastest progression is in the thoracolumbar region [43] and this could be a consequence of the thoracic cage’s presence as well as divergence of pressure between these two sites. Type of the anomaly: the worst prognosis is for a connection between the unilateral bar and the contralateral hemivertebra, but the most benevolent for progression is a complete block vertebra/incarcerated hemivertebra [3]. A completely segregated hemivertebra with healthy disc spaces around it predicts faster progression [3], and if the patient presents with the presence of more than one hemivertebra, progression may be even quicker [43]. Progression is also possible in the case of hemimetameric shifts [67], primarily in the thoracolumbar region [67,68]. And finally, a bar or a fused rib can act as a tether and push the curve progression [17,22,42,69]. Curve characteristics: the existence of two unilateral curves causes a profound malformation, whereas contralateral curves could help to equilibrate the spine [61,69,70]. If the Cobb angle’s value of the curve is smaller than or equal to 25°, then progression will be unlikely [13]. As for the unilateral unsegmented bar, development is also guided by the extent of the bar [43]. Table 1 summarizes all risk factors for rapid progression of congenital spinal deformities.

## 5. Clinical Presentation and Assessment

### 5.1. Early Diagnosis of CS

Currently, there are no targeted therapies available for CS or other congenital spinal deformities. However, early identification through genetic testing plays a vital role in predicting disease severity and potential complications—ranging from neurological deficits and respiratory issues to cardiac anomalies and orthopedic challenges. Advances in our understanding of the genetic and molecular pathways that govern spinal development during embryogenesis have opened the door to prenatal screening possibilities. Traditionally, diagnosing single-gene disorders, such as Tay–Sachs disease, fragile X syndrome, and cystic fibrosis, has relied on invasive procedures, particularly in high-risk pregnancies. In contrast, non-invasive prenatal testing (NIPT), which analyzes cell-free fetal DNA (cffDNA) circulating in maternal blood, has emerged as a safer and preferred alternative [37]. This technology holds promise for detecting congenital spinal defects earlier by leveraging insights into the genetic networks involved in vertebral development. While no genetic treatments currently exist for CS, recent breakthroughs in genetic science offer hope for future therapies. Innovations such as gene editing and regenerative medicine are being actively explored. A remarkable milestone in this field involved the successful use of CRISPR-Cas9 gene editing to treat transfusion-dependent β-thalassemia and sickle cell disease, achieved without introducing unintended genetic mutations [38]. Applying CRISPR-Cas9 to treat congenital scoliosis, however, presents unique challenges. Because CS results from structural malformations formed during early embryonic development, any corrective gene therapy would likely need to be administered in utero, during the earliest stages of gestation. Such an approach carries significant ethical, medical, and technical complexities. The permanent nature of CRISPR-induced genetic changes raises concerns about potential unintended developmental consequences [39]. Therefore, any future consideration of gene-based therapies for CS must involve a careful risk–benefit analysis. This is especially relevant given that many forms of congenital scoliosis respond well to current surgical and conservative treatments. While the potential is exciting, safety and long-term outcomes must remain at the forefront of any future clinical applications.

### 5.2. Physical Examination and Clinical Features

The presentation of congenital scoliosis is highly variable, depending on the type, severity, and location of the anomaly. Many cases are first recognized by parents or pediatricians who notice visible spinal asymmetry during infancy or routine check-ups [68]. In contrast to idiopathic scoliosis, congenital scoliosis may be present at birth or manifest very early in life, although subtle deformities can remain unnoticed until later childhood [73].

A complete physical examination should include assessment of spinal alignment in both coronal and sagittal planes, trunk balance, and clinical deformity parameters [74]. Cutaneous findings, such as dimples, hair patches, or vascular malformations overlying the spine, may suggest underlying spinal dysraphism and should prompt detailed neurological evaluation [3,6,11,12].

Neurological assessment is essential. Between 20 and 40% of patients with congenital scoliosis also present with intraspinal anomalies such as tethered cord, syringomyelia, or diastematomyelia [25,26]. Detecting these abnormalities early is critical, as neurosurgical management may be required before addressing the spinal deformity.

### 5.3. Associated Anomalies and Syndromic Conditions

Given the high incidence of comorbid anomalies, a thorough systemic evaluation is fundamental in patients with congenital scoliosis. Cardiovascular anomalies occur in 10–15% of cases, often necessitating preoperative cardiology consultation and optimization [3,6,25,26]. Genitourinary anomalies are even more common, affecting 20–30% of patients. These may include renal agenesis, horseshoe kidney, or other structural malformations, making renal ultrasound and further urological workup essential. Pulmonary complications are a major concern, especially in thoracic curves or rib anomalies [70,75]. Thoracic insufficiency syndrome, defined as the inability of the thorax to support adequate respiration or lung growth, can occur and may lead to restrictive lung disease or respiratory failure [75,76]. Early recognition and multidisciplinary management are key to optimizing long-term pulmonary development.

## 6. Diagnostic Imaging and Assessment

### 6.1. Radiographic Evaluation

The cornerstone of initial evaluation is standing posteroanterior and lateral radiographs of the entire spine, which allow assessment of curve magnitude, vertebral anomalies, and global balance [69]. Supine side-bending radiographs provide valuable information on curve flexibility, which is critical for surgical planning and anticipating achievable correction. AP and lateral plain X-rays remain the gold standard for confirming the diagnosis, classifying the anomaly, and monitoring curve progression [45]. For patients diagnosed before walking age, these radiographs can be performed in the supine position. The Cobb technique, traditionally used to measure curve severity in idiopathic scoliosis, is also considered the gold standard for angle evaluation in CS. This method involves measuring the angle between lines drawn along the upper endplate of the top vertebra and the lower endplate of the bottom vertebra [45]. The pedicle method, suggested for skeletally immature patients, has not proven to be more accurate. Since spinal growth is most rapid during the first three years of life and again during puberty, weight-bearing X-rays every six months are recommended during these critical phases to monitor curve development [26]. Plain radiographs, however, can be difficult to interpret due to overlapping anatomical structures and the small size of pediatric patients. Measurement techniques for curve severity in CS are similar to those used in idiopathic scoliosis. The Cobb method is applied to assess coronal plane deformities, while sagittal parameters are used to evaluate kyphosis or lordosis. However, interpreting these measurements requires awareness of the unique growth dynamics and rigidity typical of congenital anomalies. Serial radiographs remain essential for tracking curve progression and determining optimal timing for surgery. The frequency of follow-up should be tailored based on the patient’s age, type of anomaly, and risk of progression—with younger patients and those with high-risk anomalies requiring more frequent monitoring [3,5,6].

EOS imaging has some key beneficial aspects specific to pediatric patients, especially regarding the examination of musculoskeletal disorders. One of its key advantages is the employment of an ultra-low dose of radiation to make it safer still compared to standard radiography or CT scanning, particularly for those children who might need to be imaged repeatedly down the line. EOS also images the child with the child upright or weight-bearing to obtain more physiologically relevant data on the alignment between the spine and the lower limbs. Simultaneous anteroposterior and lateral imaging is also permitted by the system to make the scanning time shorter and to reposition the patient minimally. Also, EOS technology can create three-dimensional models from two planar ones to allow accurate measurement without the increased radiation exposure generally linked to CT-based 3D imaging. EOS imaging also has its limitations. It is a pricey technology currently not universally available within each medical center to serve all its potential users. Furthermore, since the child has to be motionless and upright during image collection, the procedure may be difficult to perform on very young children or unsteady individuals. Although EOS provides the possibility of creating 3D reconstructions, the quality of these models will be operator-dependent and dependent on the quality of the software used. EOS imaging is an excellent diagnostic tool in pediatric practice, especially for spinal deformity control, because it involves limited radiation exposure and is capable of functional weight-bearing imaging. Nevertheless, its inadequacy for soft tissue imaging, limited availability, and need for patient cooperation need to be considered when selecting the most effective imaging modalities [77,78].

The use of CT scans is limited by concerns over cost, radiation exposure, and the effect of patient posture on spinal alignment [79]. Nonetheless, 3D CT reconstructions are essential in preoperative planning. They help assess posterior spinal anatomy and can reveal up to 50% more anomalies not seen on standard X-rays [80]. Studies have shown that using 3D CT may even change the classification of certain defects—particularly in hemivertebrae—leading to modifications in clinical management. Therefore, we recommend the use of 3D CT for evaluating formation defects prior to surgical procedures such as spinal instrumentation or osteotomy. CT is best reserved for complex deformities where X-ray interpretation is challenging and for preoperative planning, rather than for routine follow-up. Additionally, lung volume calculations derived from imaging can be useful for assessing respiratory function in patients with CS [27]. Spinal dysraphism is commonly associated with CS, with a prevalence ranging from 17 to 37%, regardless of the presence of neurological symptoms [81].

When surgery is being considered—or if neurological signs are present on examination—MRI should be performed to screen for common types of dysraphism, including diastematomyelia, syringomyelia, tethered cord, dural bands, spinal cysts, and tight filum terminale [28]. Urinary tract malformations are present in up to 20% of patients with CS [82], making renal ultrasound a necessary screening tool, regardless of the presence of symptoms. If a patient is already undergoing a spinal MRI, an abdominal MRI can replace renal ultrasound to assess urinary tract anatomy [83]. Moreover, a systematic cardiac assessment is recommended for all CS patients. Echocardiography should be performed if any cardiac anomaly is suspected or as a routine precaution before scheduled procedures [6].

### 6.2. Advanced Imaging Modalities

Magnetic resonance imaging (MRI) is strongly recommended in all patients with congenital scoliosis to detect intraspinal anomalies such as tethered cord, syringomyelia, Chiari malformation, or diastematomyelia [28]. Since some anomalies may not be visible in very young children, repeat MRI may be required as the patient grows.

**Figure 2:**
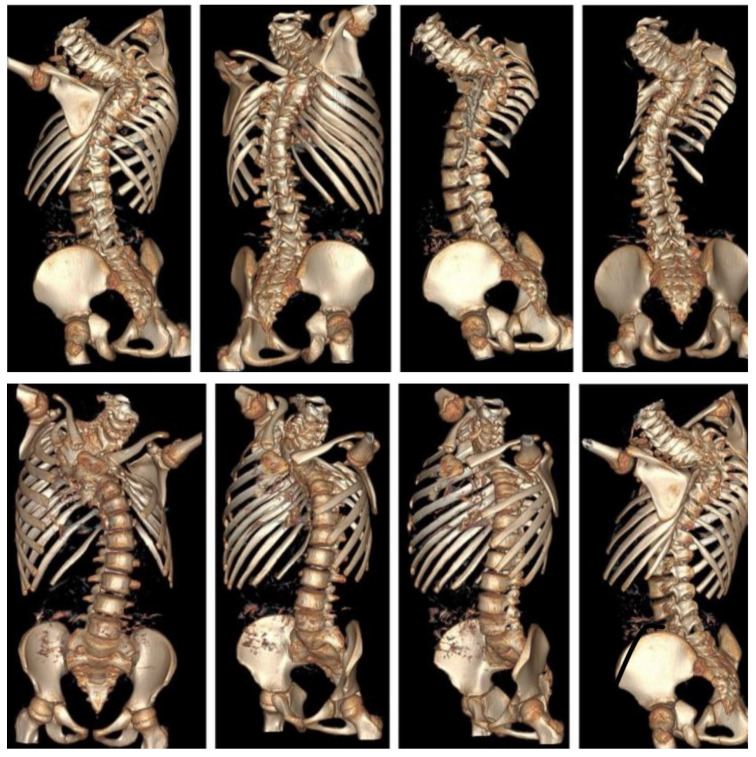
3D CT reconstruction of 5-year-old girl with congenital progressive and rigid kyphoscoliosis treated with posterior vertebral column resection at T3–T4 level.

**Figure 3:**
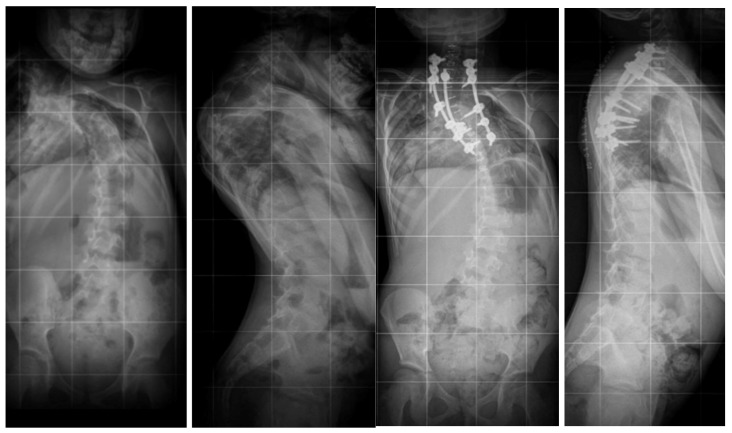
X-rays of 5-year-old girl with congenital progressive and rigid kyphoscoliosis treated with posterior vertebral column resection at T3–T4 level.

Three-dimensional computed tomography (3D CT) provides detailed visualization of complex vertebral anatomy (as shown in Figure 2), helping surgeons evaluate morphology and spinal canal dimensions. Although CT is invaluable for preoperative planning, the radiation burden must be carefully weighed, particularly in children who will need serial imaging. Postoperative control is performed using X-rays (as shown in Figure 3), unless there are postoperative doubts, in which case additional CTs and MRIs should be performed.

### 6.3. Functional Assessment

Pulmonary function testing (PFT) is essential in patients with thoracic congenital scoliosis, especially when curves exceed 50–60 degrees or rib anomalies are present [5,22]. Establishing baseline pulmonary function helps identify patients at risk of thoracic insufficiency syndrome and informs surgical decision-making [70,75]. Serial testing may be necessary to track changes and assess the effect of surgical intervention.

Quality-of-life assessments using validated instruments, such as the SRS-22 questionnaire, SRS-24, SRS-36, EOSQ-24 or the Pediatric Quality of Life Inventory, provide insight into the functional and psychosocial burden of the condition [84,85]. Incorporating these assessments into treatment planning ensures that patient and family perspectives are considered when balancing treatment risks and benefits [86,87].

## 7. Treatment Options and Decision-Making

### 7.1. Observation and Non-Surgical Management

The word ‘congenital’ is misleading since deformity may be there at birth but self-correcting curvature may or may not be found during examination [13]. Presentation time is significant, as progression is connected with spinal growth [26]. An abnormal curvature seen early in life is thus more likely to progress over time and should be treated until skeletal maturity is attained. Not all children with congenital scoliosis need emergent surgery. Close observation can be suitable for non-progressive, stable curves—more sparingly in segmentation anomalies with limited growth potential [84,88]. The decision between observation and surgical correction relies on various factors such as type and location of the anomaly and patient age, along with remaining growth potential and curve size, as well as the state of the patient with possible present systems [88]. CS encompasses a spectrum of abnormalities with different presentations as well as progression patterns. The multifaceted nature of the disease renders its management challenging as well as unpredictable. The final objective of the management, whether observation or surgery, should be prevention of the progression of the curve, as well as balanced spinal attainment. To achieve these endpoints, as highlighted earlier, the patient’s presentation age and location, as well as the nature of the deformity, are factors that are considered. As mentioned earlier, more than 70% of CS advances aggressively with surgical requirement. However, it has been established that some deformities like bloc vertebrae and wedged hemivertebrae can be treated non-operatively. Patients must be followed up periodically in the first 5 years of life (every 6 months until the age of 4 then annually prior to puberty) as well as throughout the pubertal growth spurt (every 6 months). Congenital deformities are typically stiff and rigid, hiding primary bracing. Bracing might be recommended for compensatory curves. Following consideration of the above factors, the preferred indications for corrective surgery are unilateral bar with or without contralateral hemivertebrae and over 40° deformities with higher degrees of aggressive progression, with presentation within the first 5 years of life [43,62]. Bracing has a limited role in congenital scoliosis due to the structural rigidity of vertebral anomalies [17,43]. In select cases, however, bracing can be utilized to manage compensatory curves near the primary anomaly [26,62]. The use of bracing must be individualized and mutually determined with families, weighing realistic hopes against patient tolerance [84]. In the study of Cha et al. [88] the authors investigated risk factors for developing a dominant compensatory curve—a spinal curve larger than the congenital curve—in patients with congenital scoliosis. Identifying these patients early could guide prophylactic treatment and prevent severe deformity. Using the Pediatric Spine Study Group database, researchers reviewed 307 patients aged 18 or older who had at least 2 years of natural growth without bracing or surgery. Seventeen patients (6%) developed a dominant compensatory curve, while 290 did not or had a smaller compensatory curve. A control group of 100 patients from the nondominant curve group was selected for comparison. Analysis revealed that congenital anomalies located at L4 or below were present in 18% of patients with dominant compensatory curves but 0% in controls. Additionally, anomalies at T6 or above occurred in 59% of the dominant group vs. 28% in controls. At final follow-up, patients with dominant curves had a mean congenital curve of 55° and a mean compensatory curve of 73°. The type of vertebral anomaly (wedge, hemivertebra, bar) did not differ significantly between groups. A dominant compensatory curve in congenital scoliosis is significantly associated with congenital anomalies located at T6 or above, or L4 or below. These anatomical locations may signal a higher risk of severe compensatory curves and support the use of prophylactic treatment in select patients.

### 7.2. Surgical Timing and Strategy

The timing of surgery in congenital scoliosis is critical and differs fundamentally from idiopathic scoliosis [89]. Early intervention may be required to prevent progression and to preserve growth potential, particularly in children at high risk of rapid curve advancement [86]. For surgical treatment, pediatric segmental screws or a hook system can be used [90]. The principle of “controlled growth” guides surgical planning, aiming to prevent severe deformity while allowing as much normal spinal and thoracic growth as possible [85,91,92,93,94,95].

Surgical timing depends on the rate of progression, deformity magnitude, associated anomalies, and overall patient health [84,85,86]. In general, curves that progress by more than 10 degrees per year or that reach 40–50 degrees should be considered for surgery [1,3,6,96]. Based on available scientific studies, the risk of curve progression in congenital scoliosis requiring surgical treatment is estimated to be as high as 75% [3,5,7,30].

### 7.3. Surgical Techniques

#### 7.3.1. Convex Growth Arrest and Epiphysiodesis

**Figure 4:**
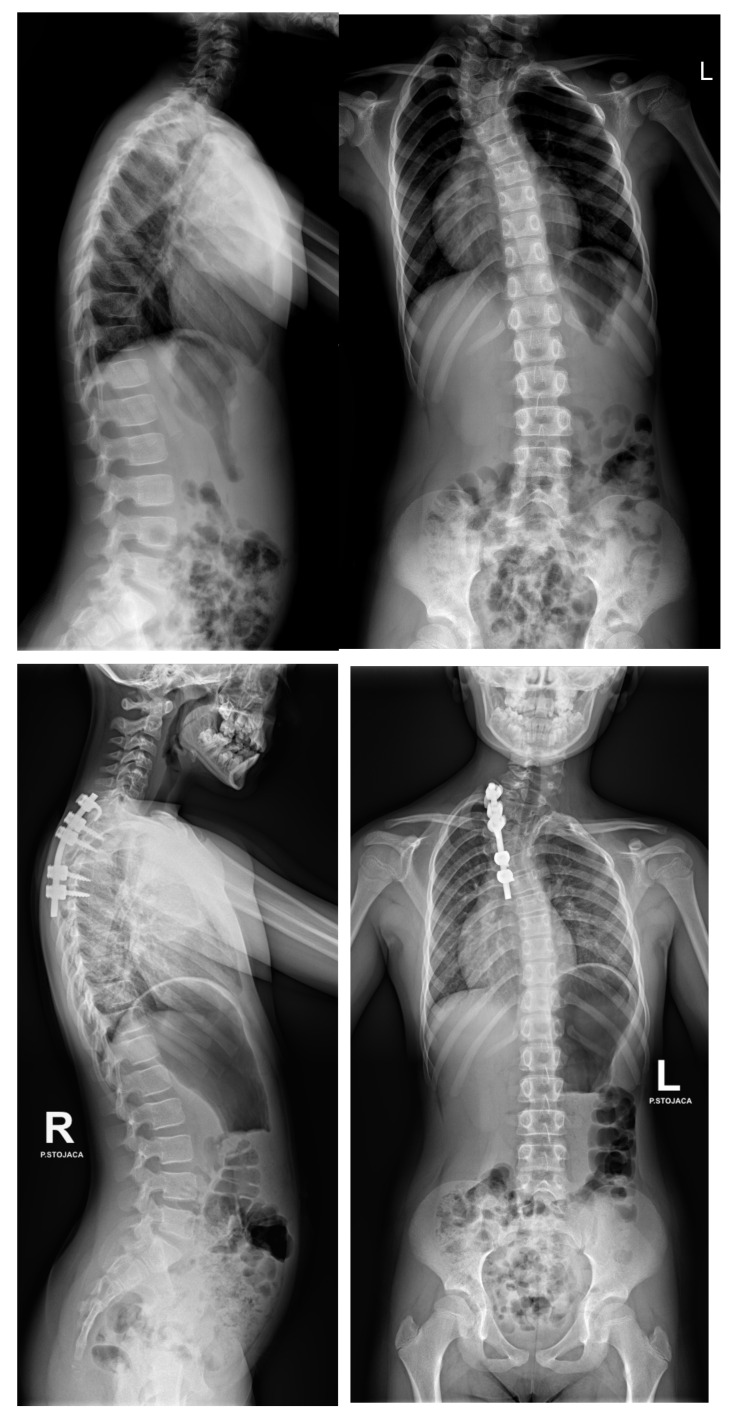
A 6-year-old girl with congenital scoliosis and kyphosis was treated with in situ arthrodesis due to multi-level segmentation defects in the spine. Given the complexity of the anomalies, this treatment technique was considered the least risky and provided satisfactory outcomes. X-rays are shown from before surgery and at the 4-year follow-up.

Convex growth arrest (convex epiphysiodesis) involves fusing the convex side of the curve while allowing the concave side to grow, promoting gradual correction. It is best suited to young patients with moderate curves (30–60 degrees) and significant growth remaining [68,97]. The procedure may be performed via anterior, posterior, or combined approaches [53]. Long-term studies report effective stabilization and even spontaneous correction in appropriately selected patients [97], as shown in Figure 4.

The rationale of the procedure is to arrest the growth potential of the convex side of the curve. The same concept routinely used for deformity of growing long bones is utilized: convex hemiepiphysiodesis reduces convex side growth, but the concave curve continues to grow, with progressive deformity reduced [98]. The surgeon should excise the lateral halves of the disks and fuse the vertebrae together anteriorly and posteriorly [98]. It is necessary that the convex side possesses growth potential (a patient young enough that significant correction can happen (age < 6 years)). This means that the procedure is futile if there is a unilateral block vertebra deformity and ideal for a fully segmented hemivertebra anomaly [99]. The growth potential discrepancy brought about between the concave side and the convex side should reestablish equilibrium in the deformed segment. Ideally, this procedure takes one level above the deformity and one level below the deformity, without exposing the concave side of the curve. Some reports have shown this procedure to achieve 15° of correction, whereas some studies have shown no correction [99,100]. Researchers [68] have highlighted the unpredictable nature of the procedure, with correction rates ranging from 20% to 70% [68]. In one study [97], the authors reported good results employing this procedure, with an overall mean correction of the Cobb angle of 35.47% and a superior correction rate with patients less than 3 years of age, with the possibility of hemivertebrae with curves less than 35° [97]. Walhout et al. surveyed the ideal conditions to employ a hemiepiphysiodesis: fully segmented hemivertebrae, aged less than 5 years, and a brief segment of a maximum five vertebrae long with a less than 70° curve and without a significant kyphotic variable [101].

#### 7.3.2. In Situ Fusion

In situ fusion is usually accomplished through a posterior approach. Exposure needs to be carried out with extreme caution because omission of posterior laminar subtle defects can cause neurological injuries. Following exposure, the imaging radiograph shows the abnormal vertebrae because the hemivertebra or bar, obvious anteriorly on X-rays, might not have coexisted or have easily identifiable posterior components, with the fusion continuing one level above as well as one level below the deformity with postoperative bracing [102]. Correction with fusion is confined to 10° or to 15° with instrumentation [103]. Instrumentation can reduce bracing time as well as augment correction preoperatively with the same neurological complication rate [104]. Ruf et al. demonstrated the potential use of pedicle screws in a pediatric patient at 1 year of age [91,92]. However, correction is lost over the years because of the fusion mass bending phenomenon as well as pseudarthrosis [105]. An isolated posterior approach preserves anterior growth potential, with progression to vertebral rotation as well as the crankshaft phenomenon [106,107,108]. In another study, researchers [109] illustrated that 15% of 54 patients less than the age of 10 years of age undergoing posterior in situ spinal fusion suffered the crankshaft phenomenon. They mentioned that it was also positively connected with previous earlier surgery as well as curves > 50° [109]. An anterior release with diskectomies can achieve a stronger arthrodesis as well as prevent crankshaft phenomena [110]. Multi-level arthrodesis at a young age is recognized to restrict the development of the lungs as well as reduce their vital capacity [104]. In situ fusion is advised as a prophylactic procedure at younger patient ages with progressive non-deforming curve with angle < 40° along the long segment [110]. In the study of Lin et al. [108] the authors showed the real incidence rates and identified the risk factors associated with the crankshaft phenomenon in CEOS patients who underwent pedicle screw fixation and PSF before the pubertal growth spurt, and explored surgical strategies aimed to prevent this phenomenon. Eighty-one patients were enrolled, with a mean follow-up time of 97 months (60–192 months). The mean age was 7 years (2–10 years) preoperatively and 15 years (14–25 years) at the last follow-up. The overall incidence of the crankshaft phenomenon was 32.10%, and 46.15% patients required revision surgery. The incidence of the crankshaft phenomenon significantly increased at age 5 years and younger and number of fused segments <5. A younger age (≤5 years old) and short segmental fusion (<5 segments) can help predict the crankshaft phenomenon in thoracic CEOS patients.

#### 7.3.3. Hemivertebrectomy

Hemivertebrectomy, excision of an ectopic hemivertebra, is still the most certain cure for this type of anomaly [8]. The operation can be undertaken with a combined anterior–posterior exposure or is more commonly nowadays conducted with a posterior-only exposure, this latter procedure being found to decrease morbidity without diminishing correction [111,112,113].

**Figure 5:**
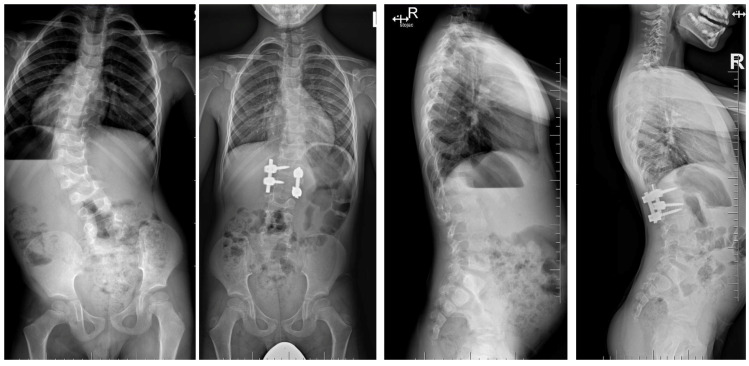
X-rays of 3-year-old boy with congenital scoliosis caused by additional hemivertebrae in thoracic and lumbar spine. Hemivertebrectomy was performed in lumbar spine from posteriori approach only with short posteriori spinal fusion. X-rays show spine before surgery and at 4-year follow-up.

**Figure 6:**
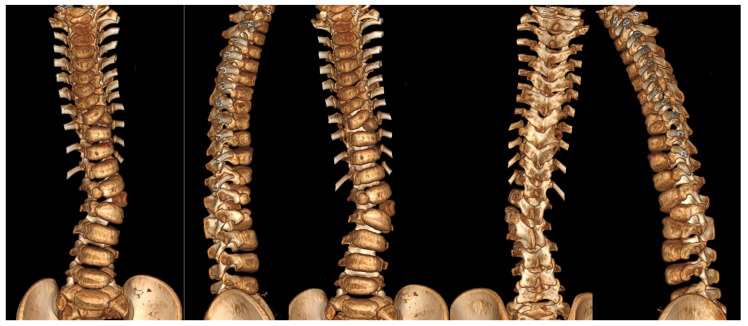
3D CT of 3-year-old boy with congenital scoliosis caused by additional hemivertebrae in thoracic and lumbar spine.

**Figure 7:**
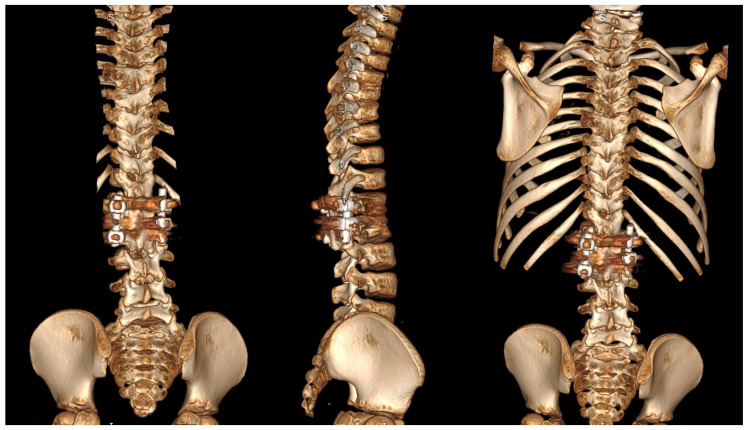
3D CT of 3-year-old boy with congenital scoliosis caused by additional hemivertebrae in thoracic and lumbar spine, who underwent hemivertebrectomy in lumbar spine from posteriori approach only with short posteriori spinal fusion. 3D CT shows spine at 4-year follow-up. It should be noted that there is also a hemivertebra in the thoracic region; however, it is stable and has not shown any progression of the curvature, so surgical treatment has not been required to date.

The procedure involves the excision of the hemivertebrae and adjoining disks with respective lamina and pedicles in the case of severe truncal imbalance [8]. Excision with posterior approach or with sequential posterior and anterior approach is required. Neurological complications are similar with both the approaches with different rates of 10–20% [114]. The posterior approach is a more difficult one, but with reduced time of surgery and hospital stay [111]. The spinal cord in the lower region is less prone to manipulation making this procedure preferable in the thoracolumbar and lumbar regions [112]. Intraoperative neuromonitoring is indispensable to minimize neurological risk [115]. Outcomes are generally excellent, with reported correction rates of 60–80% and durable long-term results when performed by experienced surgeons [113,116,117,118,119]. In the study by Zhao et al. [116], the long-term outcomes of short fusion with vertebrectomy in patients with congenital early-onset scoliosis and vertebral formation failure were evaluated. Patients who had reached skeletal maturity were followed for an average of 11.1 years. A successful outcome was defined as having a residual curve <30°, balanced spinal alignment, and no need for revision surgery. After surgery, the average spinal curve improved significantly from 38.3° to 8.9°, with some loss of correction to 17.3° at final follow-up. The success rate was 73% of patients. Importantly, coronal balance distance was identified as an independent risk factor for poor outcomes. In the report of Zhang et al. [113], the authors presented the 18-year follow-up of a 15-day-old neonate with congenital scoliosis treated using posterior-only hemivertebra bone-disc-bone osteotomy (BDBO) without internal fixation. The infant was admitted urgently due to a lumbar bulge with central ulceration and fluid discharge. Imaging revealed a fully segmented L5 hemivertebra, L3–L4 fusion, T10–T11 anterior bone bar, syringomyelia, type I diastematomyelia, tethered cord, and an open spinal meningocele. The patient underwent a combined surgical procedure with posterior-only type BDBO. Postoperative recovery was uneventful. At 18 years post-op, the patient maintained good spinal correction, coronal and sagittal balance, and no signs of pseudoarthrosis. The facet joints at L5–S1 remained well preserved, and spinal motion was satisfactory. In a study by Liu et al. [115], the researchers aimed to evaluate whether removing the intervertebral disc (IVD) adjacent to a hemivertebra (HV) affects surgical outcomes in pediatric patients undergoing hemivertebra resection. A total of 42 children with fully or semi-segmented HV were included and divided into two groups: IVD preservation and IVD removal. Postoperative correction of scoliosis was significant in both groups. However, no significant differences were found in coronal balance, sagittal plane parameters, or overall complication rates between the two groups. Importantly, preserving the IVD did not result in increased scoliosis recurrence or worse long-term outcomes. In a different study [117] there were evaluated outcomes in 22 pediatric and adolescent patients (mean age 8.3 years) with cervicothoracic scoliosis (CTS) who underwent hemivertebra resection using a modified sequential correction technique. The most commonly resected levels were T1 (31.8%) and T3 (27.6%). All patients received screw–hook hybrid constructs, with 3-rod (81.8%) and 4-rod (18.2%) systems. Significant postoperative improvements were observed in spinal alignment parameters, including cervicothoracic scoliosis angle, T1 tilt, neck tilt, clavicular angle, head tilt, and head shift, with no loss of correction at final follow-up. Complications included dural tear and iatrogenic Horner’s syndrome (each in one patient), and transient bilateral nerve root paralysis in another. Three patients experienced distal curve progression requiring revision surgery. In the study of Peng et al. [118] the authors examined long-term vertebral growth patterns and risk factors for the crankshaft phenomenon in children with congenital early-onset scoliosis (CEOS) caused by hemivertebra. The focus was on comparing spinal growth and identifying modifiable surgical risk factors following posterior hemivertebra resection (HVR) with mono-segment fusion. A retrospective analysis was conducted on 31 patients under 10 years old, treated between 2003 and 2019. At an average follow-up of 8.35 years, 29% of patients developed the crankshaft phenomenon, all showing moderate to severe vertebral rotation, compared to none in the non-crankshaft group. Although spinal dimensions increased significantly in both groups, there were no significant differences in final measurements, absolute growth, or growth rates. Multivariate analysis identified incomplete hemivertebra resection as the main risk factor. In CEOS patients undergoing posterior HVR with mono-segment fusion, crankshaft deformity is linked to incomplete resection and increased vertebral rotation. Complete resection and careful long-term follow-up are essential to reduce the risk of crankshaft progression. In another study [119], the authors developed a technique combining posterior hemivertebra extended resection with concave anterior column reconstruction. This study aimed to evaluate the outcomes of the modified posterior hemivertebra resection (MPHR) technique in older children with rigid congenital scoliosis. A retrospective analysis was conducted on 15 patients with congenital scoliosis, all of whom were over 10 years old and had less than 30% flexibility. They underwent posterior hemivertebra extended resection combined with concave anterior column reconstruction. The mean follow-up time was 2 years. The segmental curve resulted in a correction rate of 80%. The segmental kyphosis improved, resulting in a correction rate of 83%. The correction rate for the compensatory cranial and caudal curve was 59% and 66%, respectively. The MPHR provides satisfactory correction of hemivertebra deformity in older children. Figure 5, Figure 6 and Figure 7 show the outcomes of posterior hemivertebrectomy for a 3-year-old boy at 4 years of follow-up.

In the study of Xiao et al. [107], the authors retrospectively reviewed a consecutive cohort of CS patients who underwent HV resection and short fusion with a minimum follow-up of 2 years. Patients were divided into two groups based on the presence or absence of compensatory curves preoperatively: the compensatory curve group (Group C) and the non-compensatory curve group (Group NC). Furthermore, patients were categorized into progression (Group P) and non-progression (Group NP) groups based on the evolution of the coronal curve after surgery. The incidence of postoperative progression of the coronal curve was notably higher in Group C compared to Group NC, with incidences of 29.3% and 12.9%, respectively. The presence of the compensatory curve appeared to increase the likelihood of postoperative curve progression in CS patients undergoing posterior HV resection and short fusion.

#### 7.3.4. Growing Rod Systems

Growing rods have revolutionized the correction of early-onset scoliosis with the control of deformity preservation of spinal as well as thoracic growth [120]. The rationale is to maintain the growth potential of the spine with restriction of progression of the curve [121]. The ultimate goal is achieving spinal length conducive to normal function of the lungs. This procedure is applicable to long segment deformities. Interconnectors fixate the rods with hook fixation above and below the curve with local fusion [121]. Conventional systems involve repeated lengthening operations every 6–12 months and depend on growth until long-term definitive fusion [87]. Currently, the use of double-rod systems is favored since they achieve better stability as well as correction compared to single-rod systems [122].

**Figure 8:**
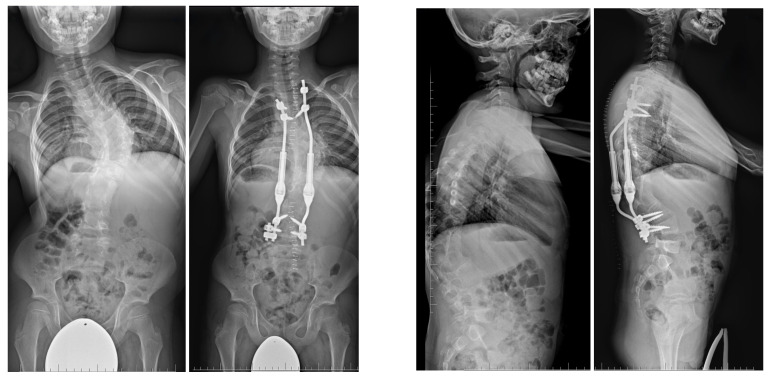
A 6-year-old boy with congenital scoliosis and mixed anomalies, treated with minimally invasive controlled growing rods (MICGRs), at 2-year follow-up after the initial surgery.

Magnetically controlled growing rods (MCGRs) represent a major innovation, allowing non-invasive lengthening via an external magnetic actuator [123,124]. Operative videos have demonstrated the technical precision required for successful MCGR placement [125]. Multicenter studies on MAGEC systems have shown significant improvements in deformity correction and spinal growth [120]. However, complications—including implant failure, internal debris extrusion, and actuator malfunction—have also been reported [126]. Some manufacturers recommend removal after two years, leading some surgeons to return to conventional rods while awaiting further refinement [87,127]. Minimally invasive controlled growing rods (MICGRs), as shown in Figure 8, similar to MCGRs, represent another evolution, aiming at reducing surgical morbidity while maintaining the benefits of controlled spinal growth [122].

In the study of Grabala et al. [87,120], among the patients there were 51 neurological, 42 syndromic, 58 idiopathic, and 10 congenital scoliosis etiologies. MCGR treatment allowed for an average correction of the curvature of 50% during the period of lengthening, while controlling any deformity and growth of the spine, with a significant increase in the T1–T12 and T1–S1 values during the observation period. MCGR treatment in EOS carries a risk of complications. While congenital and syndromic EOS often have short and less flexible curves in those groups of patients, single rods can be as effective and safe. Definitive fusion results in a mean final coronal correction between the start of MCGR treatment and after undergoing PSF of approximately 70%. The mean T1–T12 spinal height increased by 75 mm, while the T1–S1 spinal height gained a mean of 97 mm. In the study of Maccaferri et al. [128], the authors compared, in a large series of patients, the potential and limitations of the different distraction-based surgical techniques to establish the most suitable surgical approach to treat EOS. The patients included had a mean age of 7 years and a mean follow-up of 36 months. VEPTR was mainly used in congenital scoliosis (50% vs. a mean value of 25.8%) and syndromic scoliosis (42.9% vs. a mean value of 25.8%). MCGR was mainly used in idiopathic scoliosis (73.9% vs. an average value of 41.9%). TGR was mostly used in muscular neurology EOS (16% vs. an average value of 6.5%). The collected data show a similar deformity correction rate in growing rod implants in VEPTR, TGR, and MCGR. The mean curve reduction was 25.8% (95% CI (21.8–29.8) (*p* < 0.0005)). Compared with preoperative measurements, significant differences in curve magnitude correction between subgroups occurred at the final treatment measurements, when patients with MCGR had a significantly larger correction (53.2° ± 20.84 in %33.9 con DS ± 14.27) than VEPTR (27.12°± 19.13 in %19.7° ± 13.7). Different growing rod techniques are applied based on EOS etiology. While all EOS etiologies benefited from this surgical approach, congenital EOS had poorer results. Overall, MCGR has been the preferred option for idiopathic EOS and appears to be the most effective in correcting the primary curve. In the study of Glowka et al. [87], the authors of this multicenter study were to analyze the risk of complications among patients with EOS treated using magnetically controlled growing rods (MCGRs) and assess the patients’ and their parents’ quality of life after diagnosis and treatment with a minimum two-year follow-up. This study involved 161 patients who were classified according to the etiology of curvature. Implant-related complications requiring instrumentation revision were recorded in 26% of the patients. Medical complications occurred in 45% of the population. The EOSQ-24 revealed a significant improvement in average scores during the follow-up. The treatment of early-onset scoliosis with MCGRs carries 66% risks of incurring medical and mechanical complications, with 26% of patients requiring revision procedures. Children with neuromuscular scoliosis, females, and those with curvature greater than 90 degrees are at a higher risk of developing complications. Limiting the number of elective surgeries needed to prolong the instrumentation and treatment process for patients with MCGRs can greatly enhance their quality of life and satisfaction throughout the follow-up period.

#### 7.3.5. Impact on Pulmonary Development and Vertical Expandable Prosthetic Titanium Rib (VEPTR)

The relationship between congenital scoliosis and lung development is both complex and critically important for long-term outcomes [5]. Thoracic deformities, particularly when accompanied by rib anomalies or severe early-onset curves, can significantly impair pulmonary development [75]. The term thoracic insufficiency syndrome describes the inability of the thorax to support normal respiration or lung growth, a complication seen in some patients with congenital scoliosis [5,129,130].

Early surgical intervention may be necessary to preserve thoracic volume and ensure adequate pulmonary development [84]. However, extensive spinal fusion in very young children can paradoxically limit thoracic growth and compromise lung function [84]. This paradox underscores the importance of growth-preserving strategies in early-onset congenital scoliosis [131].

**Figure 9:**
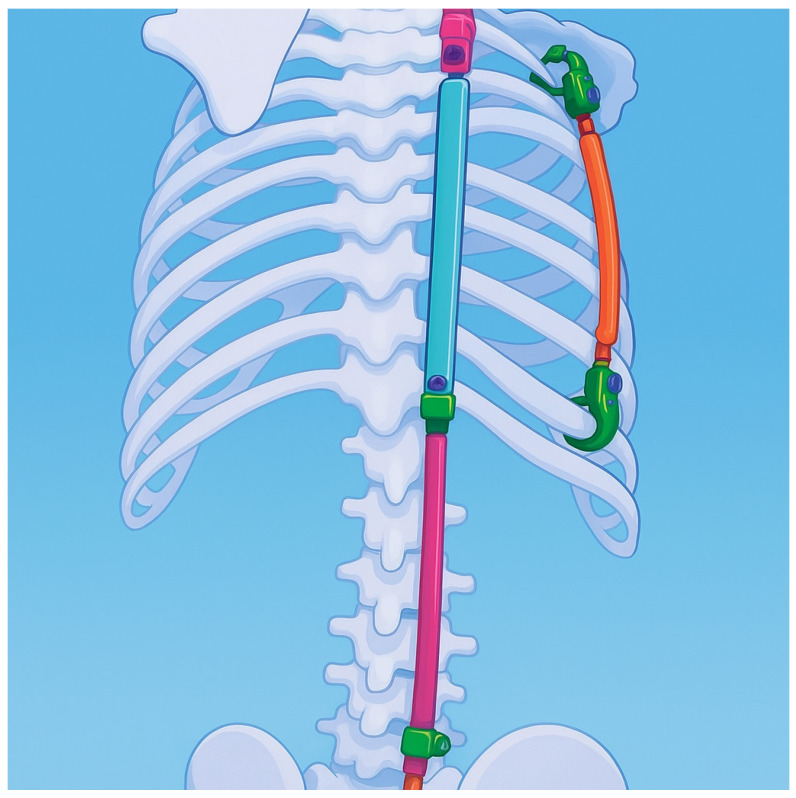
The VEPTR device for Early-Onset Scoliosis management. The Vertical Expandable Prosthetic Titanium Rib (VEPTR) is a specialized device developed to support children suffering from thoracic insufficiency syndrome (TIS), a condition commonly associated with congenital scoliosis and rib malformations. Its primary goal is to stabilize and gradually expand the chest cavity, helping the lungs develop properly and slowing down the progression of spinal curvature. Depending on a child’s specific anatomy, the VEPTR device can be attached between the ribs, from rib to spine, or from rib to pelvis. It operates using a distraction-based system, which allows the device to be lengthened as the child grows—typically through minor surgical procedures every 4 to 6 months. By increasing the space within the thorax and managing spinal deformity at the same time, VEPTR helps preserve lung function and delays the need for early spinal fusion, which can hinder growth.

The VEPTR device provides an innovative option for treating thoracic insufficiency syndrome associated with congenital scoliosis and rib anomalies [70]. By expanding the thoracic cavity, VEPTR supports lung growth while simultaneously addressing spinal deformity [75]. Similarly to growing rods, the device requires periodic lengthening to accommodate ongoing growth [132]. Figure 9 shows an animation of the VEPTR device.

Clinical studies have shown that VEPTR treatment can improve thoracic volume and pulmonary function in carefully selected patients [96]. However, the technique is not without drawbacks—it is associated with significant complication rates and typically requires multiple surgical procedures over the course of childhood [133]. When vertebral anomalies present with multiple fused or missing ribs, thoracic insufficiency syndrome can develop [134]. This condition, caused by restricted chest wall expansion, can halt lung growth and lead to severe respiratory complications—even death. To prevent such outcomes, even before symptoms arise, rib distraction techniques may be employed [5,134]. The procedure typically involves several open wedge thoracotomies, followed by the implantation of vertical expandable prosthetic titanium rib (VEPTR) devices [98]. These devices are anchored around the second and third ribs at the top, and to the lower ribs near the lumbar spine, sacrum, or pelvis. Distraction is then performed every 5–6 months along the concave side of the spinal curve to help slow its progression [98]. Studies have shown that VEPTR can improve trunk balance, Cobb angle, and spinal tilt [129,130,135]. However, pulmonary outcomes remain debated; while lung volumes and vital capacity may improve, postoperative chest wall compliance often decreases [70]. The procedure carries a relatively high risk of complications, with neurological issues occurring in around 7% of cases—most commonly brachial plexus palsy [134]. Rib and lamina fractures, infections, and other soft tissue injuries are also frequent [134]. In some cases, postoperative ossification of the device can lead to a severe reduction in chest wall mobility [134]. Given these risks, VEPTR is generally considered a last-resort treatment for thoracic complications in congenital scoliosis when more conventional approaches fail. A retrospective review [136] was conducted on nine consecutive patients with Congenital-EOS and thoracic hypoplasia treated with VEPTR at a single tertiary care center, with assessment of clinical and radiological outcomes. At mean duration of 7.4 (range 4.3–10.5) years of VEPTR treatment, the mean coronal deformity angle measured 65° preoperatively, 50° postoperatively, and 58° at final follow-up. Mean T1–S1 length (pre-op 252 mm, final follow-up 333 mm) and T1–T12 length (preop 128 mm, final follow-up 196 mm) improved by 32% at final follow-up. Mean space available for the lung was 86% (range 79–93%) preoperatively, increasing to 90% (range 85–95%) postoperatively and 97% (range 87–107%) at final follow-up. Nine children had a cumulative 17 (188%) complications comprising wound problems, infection, and device migration or prominence. In five patients who underwent definitive fusion, mean coronal deformity angle and T1–S1 length improved by 17% and 11%, respectively. VEPTR is valuable in managing EOS, particularly in patients with thoracic insufficiency syndrome. However, the need for multiple surgeries, limited correction potential, and risk of partial loss of correction make it less suitable for other cases [136].

#### 7.3.6. Growth Guidance Systems—Shilla

Shilla Growth Guidance Surgery (SGGS) represents an innovative approach to pediatric spinal deformity correction that addresses a critical challenge in pediatric orthopedics: how to correct spinal abnormalities while preserving the natural growth potential of a developing spine [137]. This technique offers a promising alternative to traditional methods by allowing continuous spinal development without requiring multiple scheduled surgical interventions. The Shilla growth guidance technique employs a sophisticated mechanical system designed to control scoliotic deformity while accommodating ongoing vertebral growth. The procedure involves the strategic placement of dual stainless-steel rods, which are secured posteriorly to the corrected spinal apex using pedicle screws. A key innovation of this approach is the incorporation of sliding pedicle screws that permit vertebral growth in both cephalad (upward) and caudad (downward) directions, while maintaining a limited fusion only at the apex of the curve.

A comprehensive analysis conducted by the Pediatric Spine Study Group examined outcomes from their multicenter database, focusing on patients who underwent SGGS with a minimum two-year follow-up period [137]. The patient cohort represented diverse etiologies of scoliosis, including neuromuscular conditions (34%), syndromic disorders (30%), idiopathic cases (29%), and congenital abnormalities (8%). The clinical results demonstrated significant therapeutic benefits. Patients experienced substantial curve correction, with major curve measurements improving from an average of 69 degrees preoperatively to 32 degrees immediately following surgery, though some correction loss occurred over time, with curves measuring 49 degrees at final follow-up. Regarding spinal growth preservation, patients achieved an average T1–S1 height increase of 7 ± 9 mm annually, with an overall height gain of 24 ± 35 mm throughout the follow-up period. An important finding from this research revealed that patient age significantly influences surgical outcomes. Children younger than seven years demonstrated an increased risk of requiring SGGS-related reoperations, highlighting the importance of careful patient selection and counseling. Interestingly, the preoperative severity of the major curve did not significantly correlate with any measured outcome, suggesting that the technique’s effectiveness is relatively independent of initial deformity magnitude. The analysis indicated a reoperation-free survival rate of 50% at 3.5 years post-surgery. Despite the elevated complication risk in younger patients, the researchers emphasized that SGGS continues to provide substantial benefits for this vulnerable population, offering meaningful curve correction while preserving growth potential [137]. A subsequent investigation specifically examined T1–S1 growth patterns in patients treated with the Shilla technique [138]. This study, which included 20 patients with a mean surgical age of 5.7 years, encompassed various diagnostic categories: syndromic conditions (*n* = 9), neuromuscular disorders (*n* = 5), idiopathic scoliosis (*n* = 3), and congenital abnormalities (*n* = 3). The preoperative mean Cobb angle measured 77 degrees (range 33–111°). The growth analysis revealed mixed results. While patients experienced an immediate mean T1–S1 length increase of 51.5 mm from preoperative to postoperative measurements, the subsequent growth during the follow-up period was more modest, with a change of 21.8 mm (4.2 mm annually), representing only 36% of predicted normal growth. The study documented a substantial revision surgery rate, with 75% of patients requiring 21 revision procedures, predominantly due to implant-related complications (*n* = 26). Additionally, 40% of patients ultimately required definitive spinal fusion at an average of 5.1 ± 1.2 years following the initial guided growth surgery. These findings suggest that while Shilla provides effective initial correction, long-term management often requires additional interventions. The researchers noted that the observed T1–S1 growth represented approximately one-third of predicted normal growth and was less than one-third of growth reported in previous Shilla series, indicating potential limitations in growth preservation compared to earlier reports [138]. A multicenter case-matched comparison study evaluated Shilla outcomes against dual spine-to-spine growing rods (GR) in patients treated between 1995 and 2009 [139]. This comparative analysis revealed important differences between the two approaches. The growing rod group demonstrated superior Cobb angle improvement and greater T1-S1 length increases compared to Shilla patients. However, the surgical burden differed between groups: while growing rod patients required more planned surgeries, SHILLA patients experienced more unplanned procedures. Notably, the overall complication rates were statistically similar between the two treatment modalities [139]. Recent research has also examined the performance of titanium-based growth guidance systems, with one study representing the largest successful investigation of titanium-made growth guidance systems to date [140]. The researchers concluded that these systems provide effective and stable correction while preserving spinal growth capacity over a minimum two-year follow-up period. The complication rates were deemed acceptable and comparable to other growth-friendly surgical techniques, supporting the continued development and refinement of these approaches [140]. The collective evidence regarding Shilla Growth Guidance Surgery presents a nuanced picture of its clinical utility. While the technique successfully addresses the fundamental challenge of correcting spinal deformity while preserving growth, it also presents unique challenges, particularly regarding revision surgery rates and growth achievement compared to normal developmental patterns. The age-dependent risk profile suggests that careful patient selection and thorough preoperative counseling are essential components of successful treatment planning. These findings contribute to the evolving understanding of growth-friendly spinal surgery techniques and provide valuable insights for clinicians managing pediatric spinal deformities. As surgical techniques and implant technologies continue to advance, ongoing research will be crucial for optimizing outcomes and minimizing complications in this vulnerable patient population.

#### 7.3.7. Posterior Vertebral Column Resection (PVCR)

**Figure 10:**
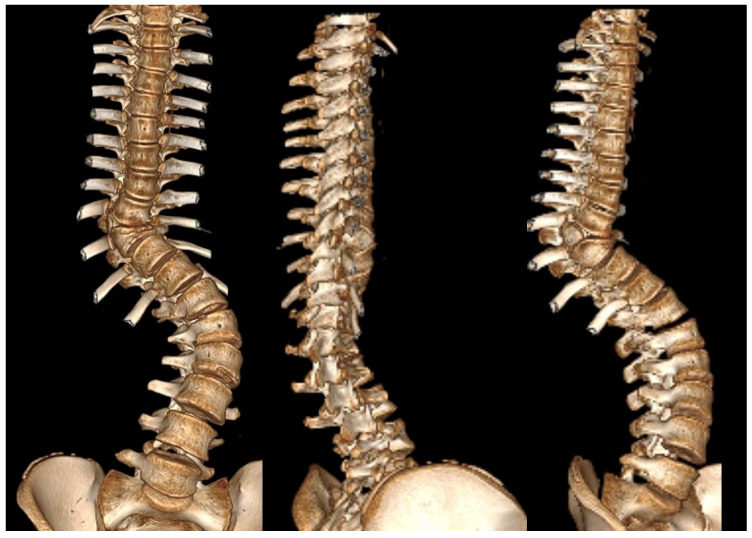
3D CT of 17-year-old boy with congenital scoliosis and insulin-treated diabetes, who underwent P-VCR T10 (posterior-only vertebral column resection of T10).

**Figure 11:**
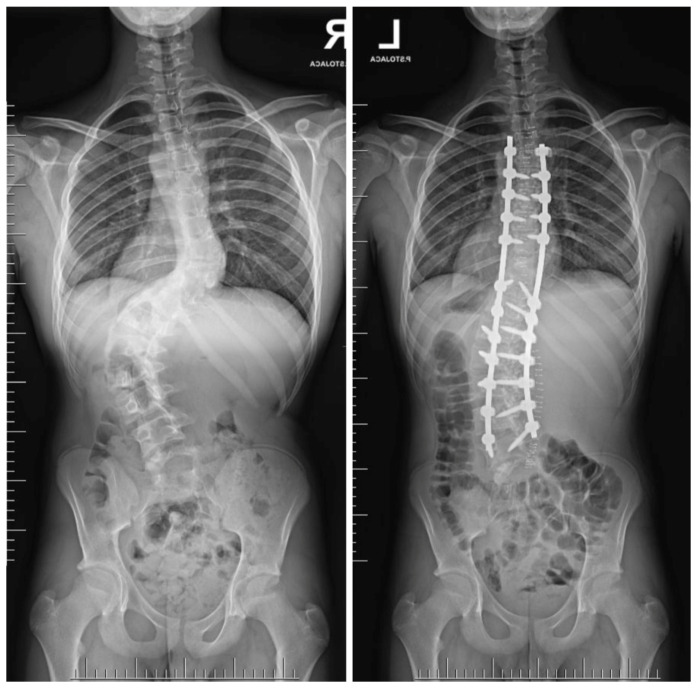

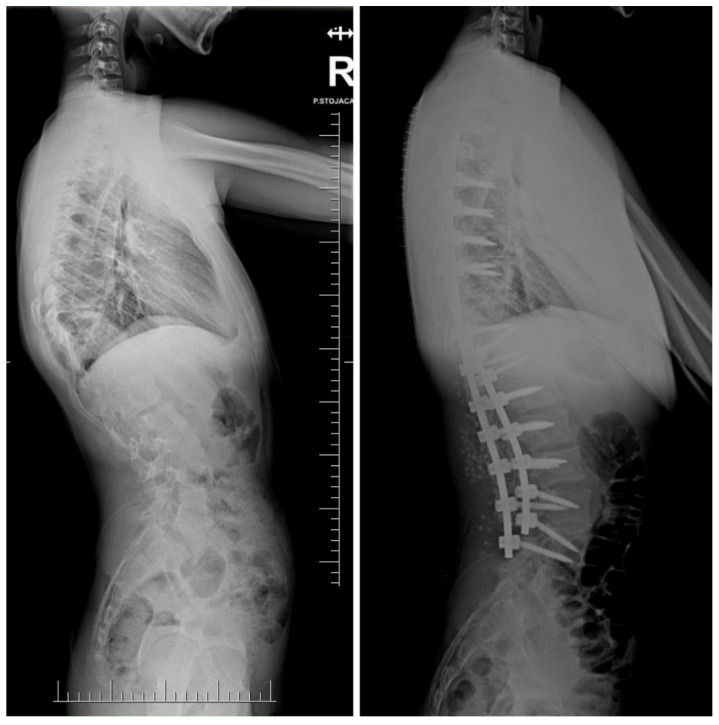
Radiographs (AP and lateral) of 17-year-old boy with congenital scoliosis and insulin-treated diabetes, who underwent P-VCR T10 (posterior-only vertebral column resection of T10).

**Figure 12:**
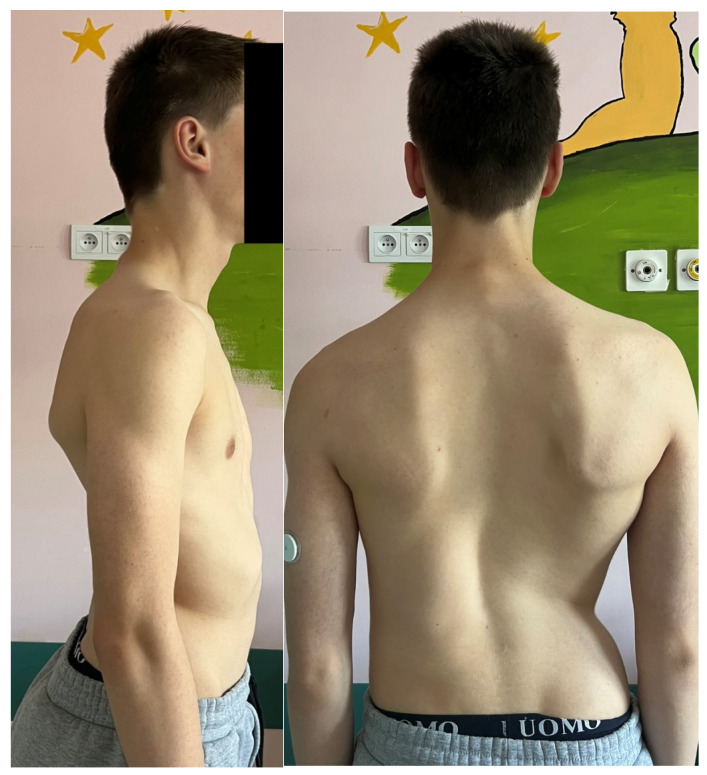
Clinical pictures for comparison before surgical treatment of 17-year-old boy with congenital scoliosis and insulin-treated diabetes, who underwent P-VCR T10 (posterior-only vertebral column resection of T10).

**Figure 13:**
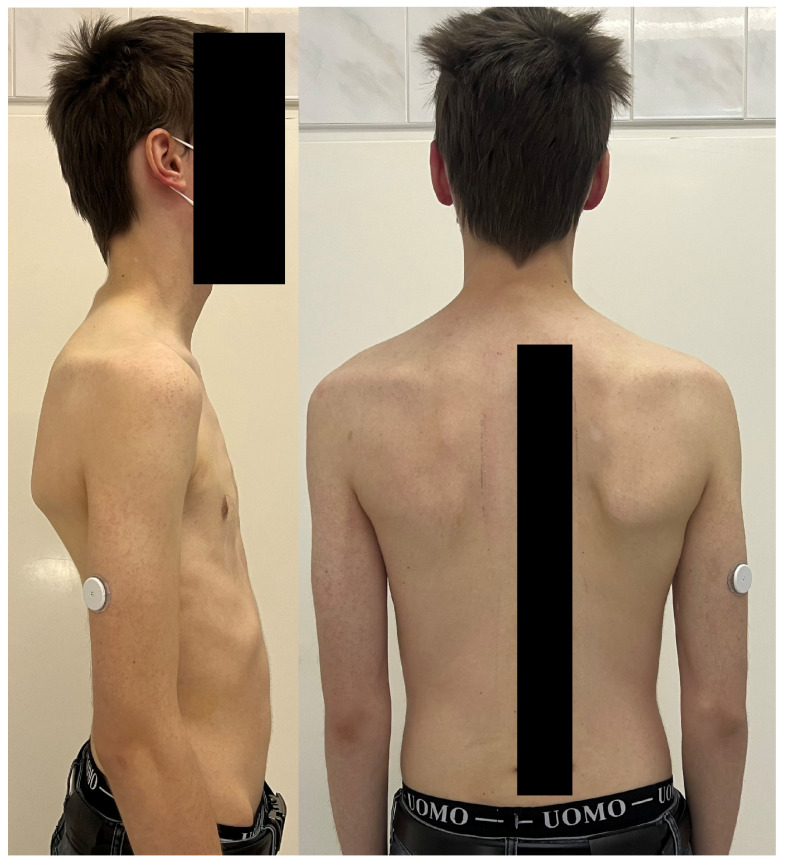
Clinical pictures for comparison after underwent surgical treatment of 17-year-old boy with congenital scoliosis and insulin-treated diabetes, who underwent P-VCR T10 (posterior-only vertebral column resection of T10).

Vertebral column resection (VCR) represents a sophisticated reconstructive procedure that involves the complete removal of vertebral elements, including the vertebral body and posterior structures such as lamina, transverse processes, and associated ribs [141]. This complex intervention can be performed through either a posterior-only approach or combined anterior–posterior techniques, serving as a salvage option for patients with rigid spinal curves, neurological complications, or failed conventional treatments [3,5]. The primary objective of this reconstructive osteotomy is to restore optimal trunk alignment through spinal shortening and removal of previous fusion masses. Posterior vertebral column resection (PVCR) has emerged as the most powerful corrective technique for severe, rigid spinal deformities, particularly congenital kyphoscoliosis [142]. By removing one or more vertebrae through a posterior approach, surgeons can achieve substantial improvements in both coronal and sagittal spinal alignment [94,143,144,145]. Figure 10, Figure 11, Figure 12 and Figure 13 present an example of this surgical technique—a 17-year-old-patient with congenital scoliosis treated with posterior vertebral column resection.

Contemporary case series demonstrate impressive corrective potential, with reported outcomes showing average Cobb angle corrections ranging from 46% to 73.6% depending on patient age and deformity characteristics [146,147,148]. A notable study from the Rizzoli Orthopedic Institute examined eight pediatric patients with severe spinal deformities, achieving a remarkable mean correction of 64% (Cobb angle reduction from 86.3° to 22.4°) with an average procedure duration of 337.4 min [147]. Age-related analysis reveals important clinical patterns. A comprehensive study of 87 patients categorized into pediatric (0–12 years), adolescent (13–19 years), and adult (>20 years) cohorts demonstrated that pediatric patients achieved the highest correction rates (73.6% coronal, 72.3% sagittal) despite requiring more extensive procedures [148]. This superior correction capacity in younger patients likely reflects the excellent compliance and adaptability of the developing spinal cord. Neurological safety remains a paramount concern in VCR procedures. Advanced intraoperative monitoring techniques, including motor and sensory evoked potential monitoring, have significantly enhanced procedural safety [147,149]. A study of 23 patients with rigid congenital deformities reported no permanent postoperative sensory or motor deficits, although temporary neuromonitoring changes occurred in two patients [149]. However, neurological complications remain a significant consideration. A prospective multicenter study of 286 pediatric patients revealed that new neurological deficits (NND) occurred in 9.4% of cases immediately postoperatively [150]. Encouragingly, the majority of these deficits resolved over time, with 21 patients demonstrating normal neurological function by the 2-year follow-up, emphasizing the importance of patient counseling regarding recovery expectations.

The complication profile of VCR varies significantly with patient age and surgical complexity. Pediatric patients, despite achieving superior correction rates, face unique challenges including higher blood loss percentages (201.9% of total blood volume) and longer operative times [148]. Common complications include sagittal decompensation (the most frequent complication), infection, and temporary neurological deficits [147,149]. Signal changes on postoperative imaging occur more frequently with VCR compared to alternative procedures, with one comparative study showing signal changes in 4 of 6 VCR patients versus 1 of 26 hemivertebrectomy patients [151]. This finding underscores the importance of careful patient selection and comprehensive preoperative planning. Recent research has explored less invasive alternatives to three-column osteotomies. A comparative study of 49 patients demonstrated that multiple Ponte osteotomies achieved comparable correction rates (54.1%) to hemivertebrectomy/VCR procedures (54.4%) while potentially reducing neurological risks [151]. Additionally, preoperative halo-gravity traction combined with posterior spinal fusion has shown promise in achieving satisfactory correction (47%) of severe deformities without requiring VCR [152]. The decision to proceed with VCR requires careful consideration of multiple factors, including deformity severity, patient age, physiological status, and presence of comorbidities. Pediatric patients with congenital vertebral and cardiac abnormalities require particularly cautious evaluation and comprehensive preoperative preparation [148]. The procedure should only be performed by experienced surgeons in specialized centers equipped with advanced neuromonitoring capabilities [132,153]. Larger preoperative kyphoscoliosis or focal kyphosis angles have been identified as significant risk factors for postoperative sagittal imbalance, highlighting the importance of thorough preoperative assessment and surgical planning [149].

**Figure 14:**
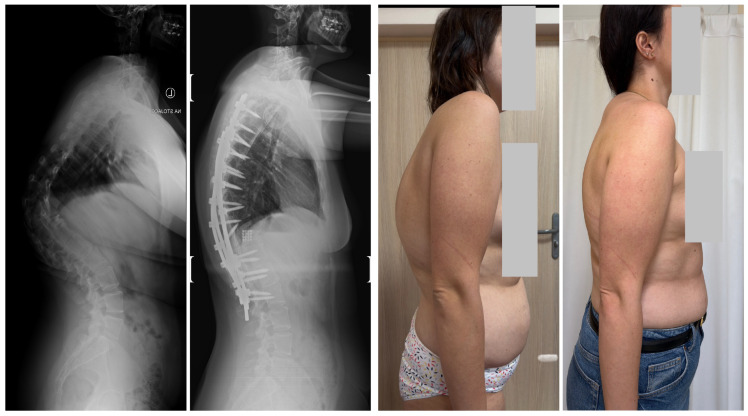

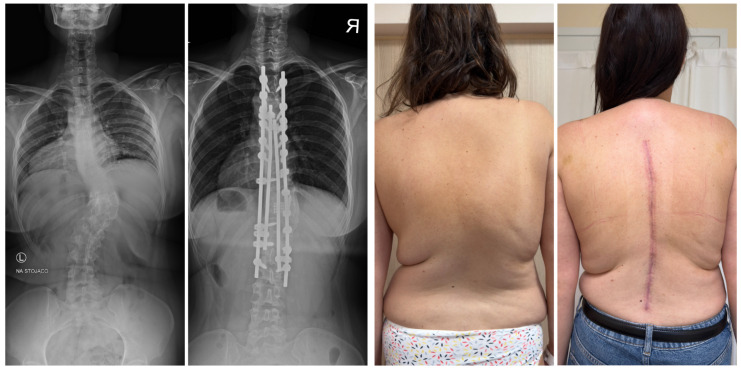
A 28-year-old woman, treated with posterior vertebral column resection because of congenital scoliosis which was not treated during adolescence.

The evolution of VCR techniques, including advances in posterior-only approaches and improved neuromonitoring, has enhanced both safety and consistency of outcomes [144,145]. While VCR remains a powerful tool for severe spinal deformities with correction rates of 60–80%, the development of alternative techniques that achieve comparable outcomes with reduced morbidity represents an important area of ongoing research [29,151]. The high recovery rate of neurological deficits over time provides reassurance for both patients and families, though the initial occurrence of such complications emphasizes the need for comprehensive informed consent and postoperative monitoring protocols [93,94,95,150]. Vertebral column resection continues to serve as an invaluable option for patients with severe, rigid spinal deformities, particularly in the pediatric population where superior correction rates can be achieved. While the procedure carries inherent risks, including neurological complications and significant blood loss, careful patient selection, advanced surgical techniques, and comprehensive perioperative care can optimize outcomes. The development of alternative approaches that maintain corrective efficacy while reducing morbidity represents a promising direction for the future management of complex spinal deformities. Figure 14 presents a 28-year-old woman, treated with posterior vertebral column resection because of congenital scoliosis.

## 8. Complications and Risk Management

### 8.1. Surgical Complications

The surgical correction of congenital scoliosis represents one of the most challenging procedures in pediatric orthopedics, carrying inherent risks that demand meticulous preoperative planning and intraoperative vigilance. Among the various complications that may arise, neurological deficits remain the most feared and clinically significant concern for both surgeons and families. Permanent neurological complications occur in approximately 1–5% of major corrective procedures, with rates varying depending on the complexity of the deformity and the extent of surgical intervention [154]. The elevated risk profile in congenital scoliosis compared to idiopathic cases stems from several interconnected factors. Patients with congenital spinal anomalies frequently present with associated intraspinal abnormalities, including diastematomyelia, syrinx formation, tethered cord syndrome, or Chiari malformations, which significantly increase the vulnerability of neural structures during surgical manipulation [87,93,146]. Additionally, the complex three-dimensional nature of congenital deformities often necessitates more aggressive corrective maneuvers, further amplifying the potential for neurological injury. The implementation of comprehensive intraoperative neuromonitoring has revolutionized the safety profile of major spinal deformity surgery and has become the standard of care in specialized centers [154]. This technology employs continuous monitoring of motor and sensory evoked potentials, providing real-time feedback regarding spinal cord function throughout the procedure. However, the reliability and interpretation of neuromonitoring signals can be compromised in patients with pre-existing spinal cord anomalies, requiring surgeons to exercise heightened caution and potentially modify their surgical approach based on baseline neurological function [155]. Beyond neurological complications, surgeons must also contend with a spectrum of other perioperative risks [156]. Surgical site infections occur with variable frequency depending on patient factors, surgical duration, and institutional protocols, with some series reporting rates ranging from 2 to 10% in complex spinal procedures. Implant-related complications present another significant concern, encompassing hardware failure, screw loosening or pull-out, rod fracture, and pseudarthrosis formation [133]. The risk profile escalates dramatically with more extensive procedures, such as posterior vertebral column resection (PVCR), which carries the highest complication rates due to the magnitude of bony resection and reconstruction required [146].

### 8.2. Long-Term Complications

The long-term management of patients with surgically treated congenital scoliosis requires ongoing vigilance for delayed complications that may emerge years or decades following the initial intervention. Adjacent segment degeneration represents a particularly concerning late complication, occurring when the fusion of spinal segments transfers increased mechanical stress to neighboring mobile segments, potentially leading to accelerated degenerative changes and the development of new deformities [85]. This phenomenon is especially problematic in pediatric patients, who must live with the biomechanical consequences of their surgical treatment for many decades, making the preservation of spinal motion segments a critical consideration in surgical planning [157]. The challenge of maintaining spinal mobility while achieving adequate correction has led to the development of growth-preserving techniques, such as growing rod systems. While these innovative approaches successfully maintain spinal length during the critical growth period, they introduce their own unique complication profile. Growing rod patients face an increased risk of implant failure due to the repetitive loading cycles over extended periods, with metal fatigue potentially leading to rod fracture or connector failure [133]. Recurrent infections at surgical sites represent another significant concern, particularly given the need for multiple revision procedures throughout the growth period. The concept of the “law of diminishing returns” describes a well-documented phenomenon in growing rod management, whereby each subsequent lengthening procedure yields progressively smaller gains in spinal height [127]. This occurs due to the development of scar tissue around the implants and the gradual stiffening of the spine over time, ultimately limiting the effectiveness of the distraction mechanism.

**Figure 15:**
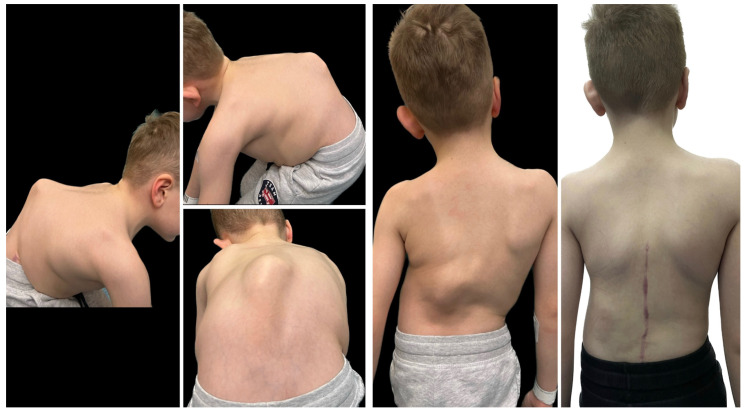

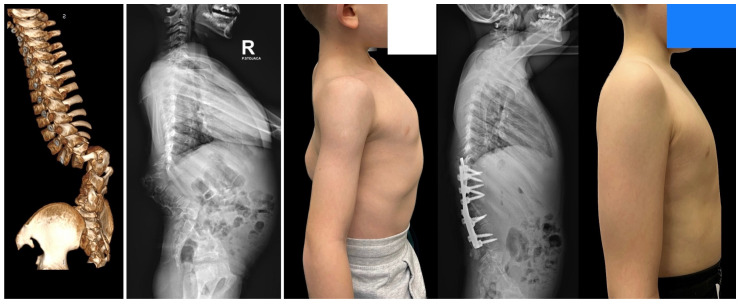
Five-year-old boy, Chiari 2, spinal cord tethered, syrinx, and rare congenital kyphoscoliosis with dislocation without neurological deficits. P-VCR was performed with short posterior spinal fusion bone-on-bone. At the 1-year follow-up, revision surgery was performed because of a broken right rod. At the 2-year follow-up, a second revision surgery was performed because of a broken right rod. At 3.5 years of follow-up, no problems or complications were noted.

Additional implant-related complications that may develop over time include screw pull-out from osteoporotic or dysplastic bone, rod breakage due to fatigue failure, and the development of pseudarthrosis at fusion sites, as illustrated in Figure 15. These complications often necessitate complex revision procedures, which carry their own inherent risks and may require more extensive surgical approaches than the original intervention. The cumulative burden of multiple procedures not only increases the overall complication risk but also impacts the patient’s quality of life and places significant emotional and financial strain on families throughout the treatment journey.

## 9. Outcomes and Prognosis

Radiographic outcomes following surgery for congenital scoliosis are generally favorable. Most studies report significant correction in both coronal and sagittal planes [74]. The degree of correction largely depends on the surgical technique used, with hemivertebrectomy typically achieving the most substantial and lasting results [8].

Maintaining this correction over time is especially important in growing children. Long-term follow-up studies show that most patients retain their initial correction, although some loss may occur, particularly when fusion is incomplete or improperly executed [55,65]. This challenge is discussed in more detail in the treatment technique sections, where the importance of choosing an appropriate surgical approach to ensure long-term spinal stability is emphasized.

Functional outcomes include pulmonary function, physical activity levels, and overall quality of life [84]. Early surgical intervention has been shown to preserve lung capacity and reduce the risk of thoracic insufficiency syndrome [84,87,158]. However, extensive spinal fusion in very young children may lead to long-term limitations in mobility and functional independence [86]. Despite these concerns, quality of life is generally reported as good following successful treatment. Patients often experience improved function and express high levels of satisfaction [85]. Still, the need for multiple procedures, along with postoperative complications, can place a considerable physical and emotional burden on both patients and their families [133].

A diagnosis of congenital scoliosis often introduces significant psychosocial stress for patients and their families [157]. Multiple surgeries, prolonged treatment timelines, and uncertainty regarding long-term outcomes can cause anxiety and emotional strain [133]. It is essential for healthcare providers to take a proactive role in offering psychological and emotional support throughout the treatment process [159]. Adolescents are particularly vulnerable to issues related to body image, which may persist even after visible correction of the deformity [158]. Early psychological counseling and continuous supportive care are important in promoting better psychosocial outcomes [157].

Comprehensive family counseling is crucial for informed and collaborative decision-making [159]. Families must receive clear, consistent information about the condition, treatment options, likely outcomes, and possible complications [7]. Support groups and patient advocacy organizations offer valuable resources—connecting families with others facing similar challenges, providing practical advice, and offering emotional reassurance [157,158].

## 10. Future Directions and Innovations

### 10.1. Genetic Therapy and Regenerative Medicine

Advances in genetics are opening new possibilities for congenital scoliosis care [29]. As understanding of vertebral development improves, targeted therapies may eventually emerge [30]. Gene therapy is currently being investigated in preclinical settings, while regenerative strategies—such as stem cell therapy and tissue engineering—hold long-term potential for restoring normal spinal growth and function [160].

### 10.2. Technological Innovations

Technological progress continues to reshape management. Improved imaging protocols (e.g., low-dose radiation and 3D reconstructions) enhance diagnosis and monitoring [77,78,161]. Navigation systems and robotics increase surgical precision and safety [117].

Artificial intelligence and machine learning are also being applied to deformity surgery, supporting diagnosis, treatment planning, and outcome prediction [162,163]. These tools may help standardize care and improve outcomes in the future.

## 11. Limitations of the Literature

Despite advances, the body of evidence on congenital scoliosis has notable limitations. Most studies are retrospective case series or small cohorts rather than randomized controlled trials, reflecting the rarity of the condition and ethical challenges in pediatric research. This limits the strength of evidence.

Study heterogeneity—differences in patient populations, techniques, outcomes, and follow-up—makes comparisons difficult and prevents meaningful meta-analysis. Long-term outcome data for newer surgical methods remain scarce, raising questions about durability, especially in patients treated during early childhood.

Inconsistencies in classification systems and reporting also complicate interpretation. Psychosocial and quality-of-life outcomes are often underreported, with most research focusing on radiographic results. Finally, variability in surgical techniques, implant systems, and perioperative care across institutions reduces generalizability.

## 12. Treatment Algorithm

Based on this review, here is suggested the following algorithm to be used to guide the management of CS. The most important criteria to select the best management are patient’s age, Cobb angle’s magnitude and form of the malformation, but surgical skills and experience is also important for all treatment courses. Every case of congenital scoliosis is unique, with varying patterns and rates of curve progression. Because of this variability, treatment plans must be carefully tailored to the individual patient. Drawing from both an extensive review of the literature and significant clinical experience in managing osseous-origin scoliosis, the treatment approach can be broadly outlined in a few essential steps.

The first step involves thorough diagnosis and evaluation. Initial imaging typically includes X-rays or EOS scans, and in more complex cases, may be extended to include CT or MRI to better visualize spinal deformities, identify intraspinal anomalies, and assess associated systems such as the heart or kidneys. Predicting the potential for progression is also critical at this stage. Patients with curves greater than 20 degrees, significant vertebral rotation, or skeletal immaturity should be promptly referred to a pediatric spinal specialist for further assessment.

The second step focuses on management strategies based on the magnitude of the spinal curve. For curves under 25 degrees, active observation is usually the preferred approach. These mild deformities are monitored with periodic physical exams and imaging—typically every 6 to 12 months. Close follow-up is particularly important during periods of rapid growth, such as early childhood (ages 0–5) and adolescence (ages 10–15), when scoliosis can progress quickly.

For curves ranging between 25 and 40 degrees in children who are still growing, non-surgical methods like bracing or serial casting may be indicated. Braces and casts aim to control progression and can often delay or even avoid surgery. Casting is usually used for infants and young children, with changes made every few months. Bracing is more effective at managing secondary, compensatory curves rather than correcting the primary defect. The overall goal is to maintain spinal alignment until the child reaches an age suitable for definitive surgical treatment. For these deformities are monitored with periodic physical exams and imaging—typically every 6–12 months and consider every 4–6 months for fast progressed curves.

When curves exceed 40 degrees or show rapid progression, surgical intervention is often necessary. The choice of surgical technique depends heavily on the child’s age and growth potential. In younger patients, expandable systems like growing rods or the MCGR system are often used. These allow for spinal correction while accommodating continued growth, with the MCGR system offering non-invasive lengthening through magnetic technology. In more severe cases involving thoracic insufficiency, procedures such as the Vertical Expandable Prosthetic Titanium Rib (VEPTR) are employed to create space for lung development. Hemivertebra resection may also be performed to remove malformed vertebrae and stabilize the spine through a short fusion or hybrid construction for more complex cases. For older adolescents who have reached or are nearing skeletal maturity, spinal fusion becomes the preferred treatment. This permanent correction procedure is commonly used for severe curves that have continued to progress despite conservative management.

The final step involves managing conditions often associated with congenital scoliosis. Many patients have additional anomalies in other organ systems, particularly the heart and kidneys. Because of this, care must be coordinated through a multidisciplinary team that may include cardiologists, pulmonologists, nephrologists, and orthopedic specialists. These associated conditions must be carefully evaluated and addressed before or during any surgical intervention.

**Figure 16:**
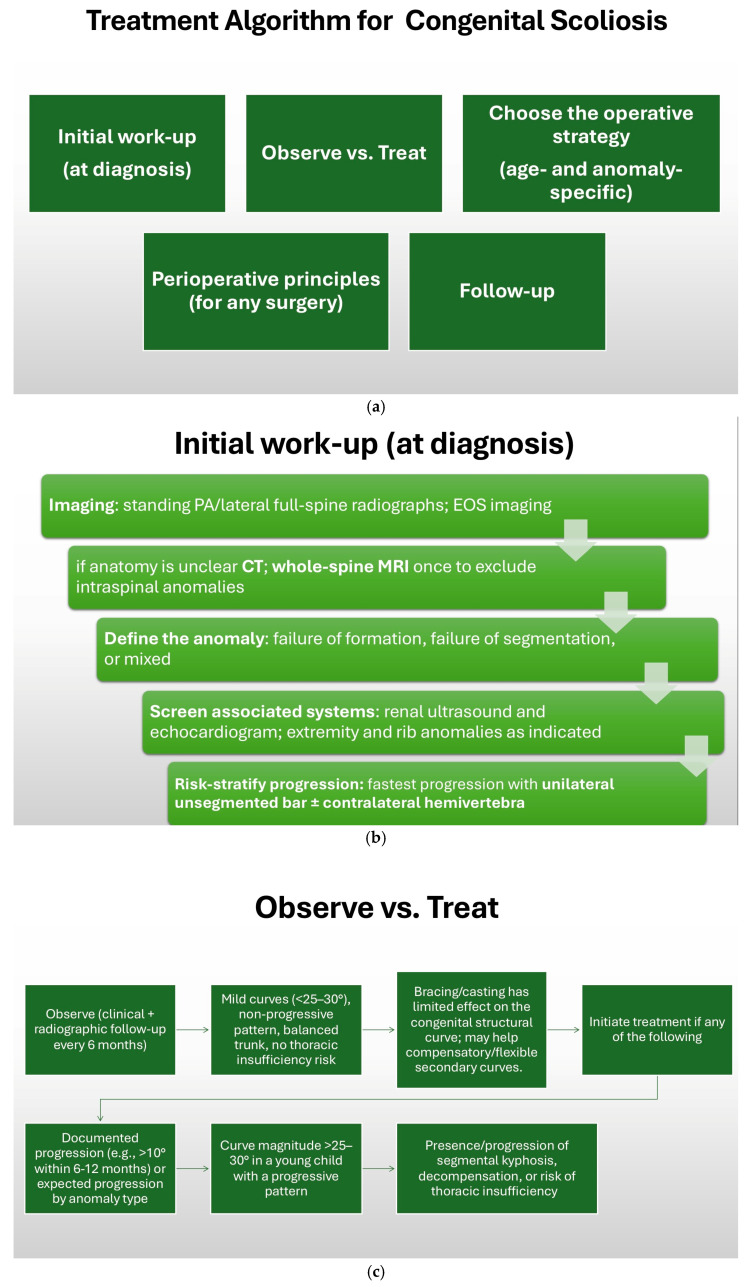

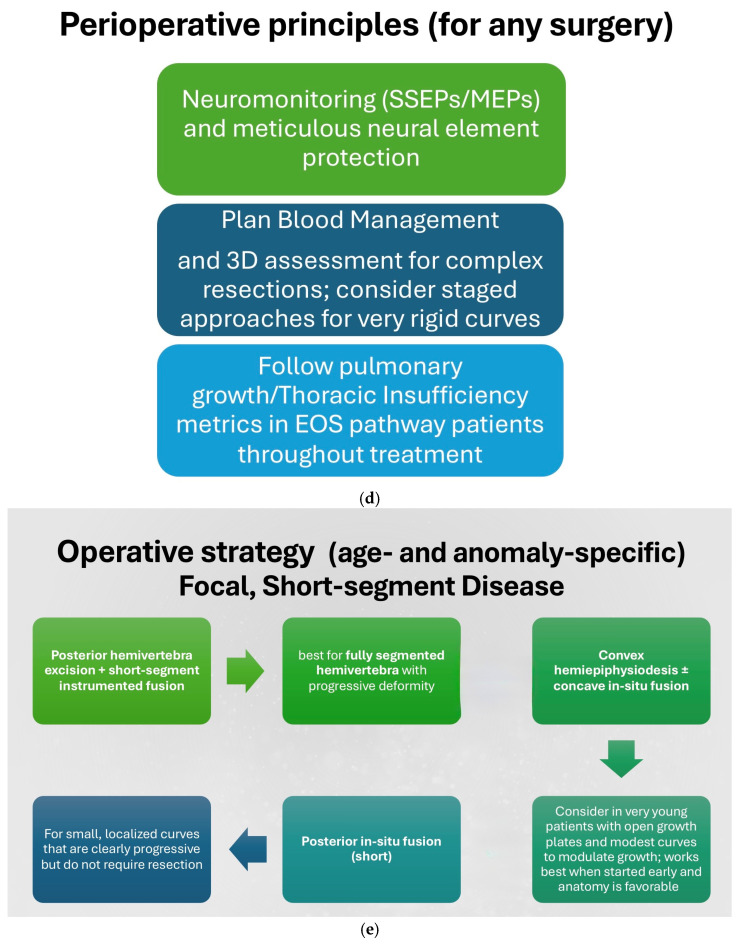

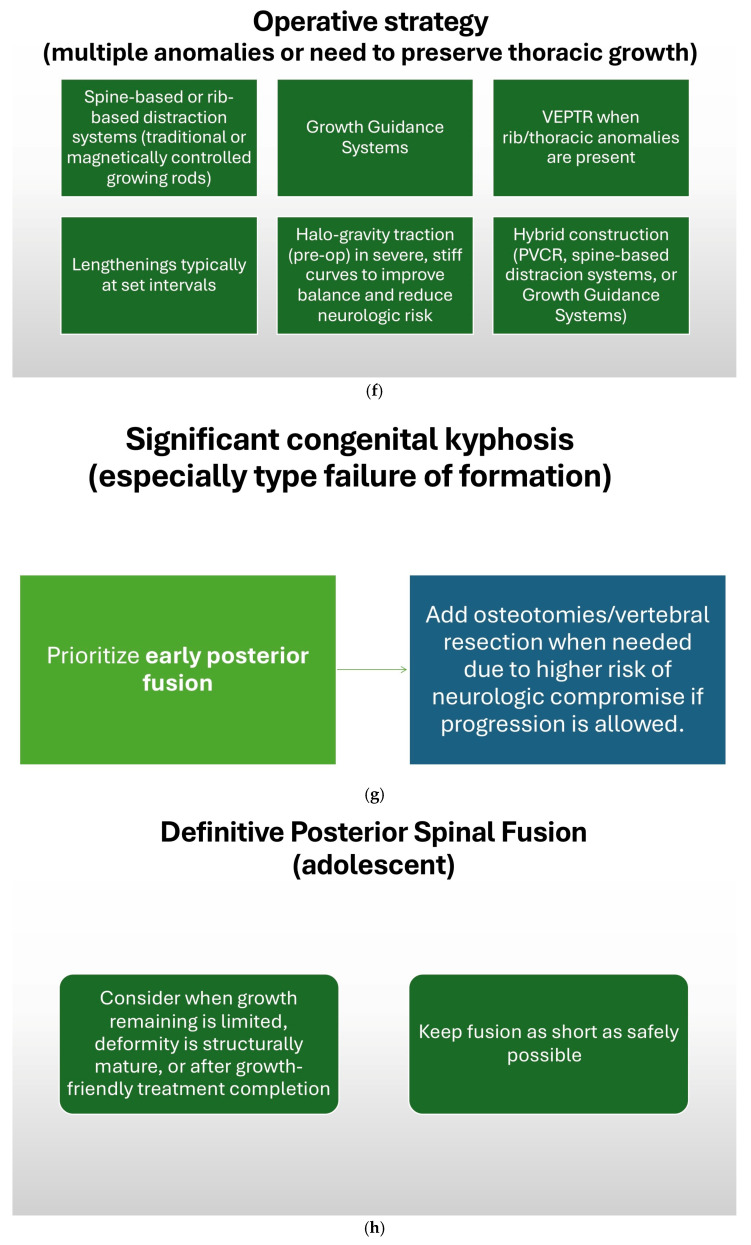

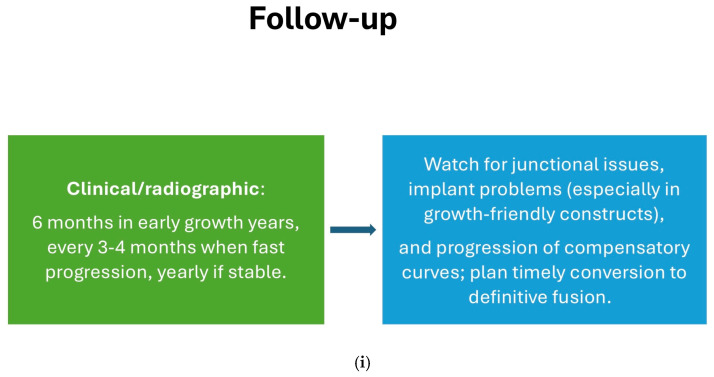
(**a**) Proposed Treatment Algorithm for Congenital Scoliosis. (**b**) Initial work-up (at diagnosis)—Proposed Treatment Algorithm for Congenital Scoliosis. (**c**) Observation vs. Treatment—Proposed Treatment Algorithm for Congenital Scoliosis. (**d**) Perioperative principles—Proposed Treatment Algorithm for Congenital Scoliosis. (**e**) Operative strategy—short segment disease. (**f**) Operative strategy—multiple anomalies. (**g**) Operative strategy—congenital kyphosis. (**h**) Operative strategy—Posterior Spinal Fusion. (**i**) Follow-up strategy.

Conclusion of this algorithm, nearer follow-up should be practiced at younger age, advancing techniques should be favored until skeletal maturity and final fusion techniques should be practiced after the 10 years of age. Proposed treatment algorithm is presented in Figure 16a–i.

## 13. Conclusions

Congenital scoliosis represents a broad clinical spectrum—ranging from stable, well-compensated deformities with minimal risk of progression to severe, rapidly advancing curvatures. It is essential to recognize the high prevalence of associated intraspinal anomalies in patients with CS. Non-surgical management is often ineffective in this population, and surgical approaches must be carefully tailored to factors such as the patient’s age, the severity of the curve, and the specific type of vertebral malformation. CS remains one of the most complex and demanding spinal conditions in pediatric orthopedics. Its treatment requires a nuanced approach that considers not only the spinal anomaly itself, but also the child’s growth potential and the broader familial and developmental context. Optimal management calls for collaborative decision-making led by pediatric spine specialists within a multidisciplinary framework. This includes coordination with genetics, cardiology, pulmonology, and psychosocial services to address the full scope of patient needs. Early diagnosis and appropriately timed interventions are key to preventing severe progression and achieving the best long-term outcomes. However, each case of CS is unique, and individualized care planning remains paramount. Innovations with connections such as refined hemivertebrectomy, magnetically controlled growing rods, and posterior vertebral column resection have expanded the treatment armamentarium, though each comes with unique risks and limitations. Ongoing research into genetic mechanisms, predictive models, and long-term functional outcomes will be critical to advancing care. Ultimately, successful management depends on balancing structural correction with preservation of growth, while addressing the broader medical and psychosocial needs of both patients and their families and surgical skills and experience of surgeon.

## Figures and Tables

**Table 1:** Risk factors for rapid progression.

Risk Factor	Details
Type of anomaly	- Multiple anomalies and mixed malformations increase progression risk [66] - Worst prognosis: unilateral bar with contralateral hemivertebra [3,6,30] - Most benign: complete block vertebra/incarcerated hemivertebra [3,6,30] - Fully segmented hemivertebra with healthy disc spaces predicts faster progression [3,6] - Presence of more than one hemivertebra increases progression rate [35,56,71] - Hemimetameric shifts, especially in thoracolumbar region, may lead to progression [56,67,72] - Bone bar or fused ribs may act as tethers and promote curve progression [35,42,56]
Apex location	- Upper thoracic curves: slowest progression - Mid-thoracic: faster progression - Thoracolumbar region: greatest progression, possibly due to thoracic cage influence and pressure divergence [43]
Patient’s age	- Greatest progression before age 5 and during adolescent growth spurt (ages 11–14) [35] - Curves visible before age 10 = poorer prognosis due to higher growth potential [30,61] - Deformities obvious in early infancy = worst prognosis [3,6,42]
Curve characteristics	- Two unilateral curves cause significant malformation. - Contralateral curves may help balance the spine [56,61] - Cobb angle ≤ 25° → unlikely progression [13] - Progression of a unilateral unsegmented bar also depends on its extent [23,43,56]
Growth asymmetry	- Progression linked to asymmetric growth between convex and concave sides, with most growth on the convex side [71]
Additional notes	- Progression occurs on “normal” disc spaces; fused segments do not progress [3,26] - Progression velocity depends on age, apex location, anomaly type, and curve characteristics [3,26]
Additional progression stats	50% of curves progress rapidly 25% progress slowly 25% do not progress at all [26]

## Data Availability

Data are contained within the article.
